# Analgesia and sedation in premature infants receiving invasive ventilation: a systematic scoping review

**DOI:** 10.1038/s41390-025-04441-y

**Published:** 2025-11-20

**Authors:** Fiona Moultrie, Xavier Durrmeyer, Gerbrich E. van den Bosch, Manon Tauzin, Jean Michel Roué, Emma Olsson, Maria M. Cobo, Luke Baxter, Samyuktha Iyer, Aomesh Bhatt, Sinno H. P. Simons, Rebeccah Slater

**Affiliations:** 1 Department of Paediatrics, University of Oxford, Oxford, UK; 2 Neonatal Intensive Care Unit, Centre Hospitalier Intercommunal de Créteil, Créteil, France; 3 Faculté de Médecine de Créteil, IMRB, GRC CARMAS, Université Paris Est Créteil, Créteil, France; 4 Department of Neonatal and Pediatric Intensive Care, Division of Neonatology, Erasmus MC–Sophia Children’s Hospital, Rotterdam, The Netherlands; 5 Department of Neonatal and Pediatric Critical Care, Brest University Hospital, Brest, France; 6 Faculty of Medicine and Health, School of Health Sciences, Örebro University, Örebro, Sweden; 7 Department of Pediatrics, Faculty of Medicine and Health, Örebro University, Örebro, Sweden; 8 Colegio de Ciencias Biologicas y Ambientales, Universidad San Francisco de Quito USFQ, Quito, Ecuador

## Abstract

**Background:**

Premature neonates often require mechanical ventilation during intensive care. However, there is a lack of clinical consensus on the provision, type, and dosage of analgosedatives. The purpose of this scoping review is to assess the risks and benefits of providing analgesic and sedative drugs to ventilated premature infants.

**Methods:**

We sourced primary empirical research reporting outcomes related to the use of pharmacological analgesics and sedatives in ventilated premature infants. We included articles published in any language in peer-reviewed journals before February 2024 from MEDLINE, Embase, Web of Science, Cochrane Library, and Google Scholar databases. We present the overall study characteristics, and the reported risks and benefits of analgosedatives within drug sub-groups.

**Results:**

80 studies were included in the scoping review. Morphine was the most studied drug (39 studies), followed by fentanyl (19 studies). Midazolam (8 studies) and dexmedetomidine (3 studies) were the most frequently studied sedatives. Analgesic efficacy was more consistently reported for fentanyl than morphine. The sedative effect of opioids was rarely assessed. Respiratory, cardiovascular, gastrointestinal, neurological and neurodevelopmental risks were unclear for all opioids. Alternative synthetic opioids and midazolam appear to be associated with significant risks in the absence of clear benefits. Dexmedetomidine shows encouraging but limited results and merits further investigation as an opioid-sparing adjunct.

**Conclusion:**

At present, fentanyl appears to have the best efficacy and safety profile for analgosedation in this patient population. This scoping review will support clinicians in their analgosedative management of ventilated premature infants and identifies research gaps and priorities.

**Impact:**

- This systematic scoping review provides a comprehensive summary of the evidence of the risks and benefits of analgesics and sedatives in ventilated premature infants.
- Although morphine is the most extensively studied and used drug, its analgesic effect has been less consistently reported than that of fentanyl.
- Sedation has rarely been assessed and dexmedetomidine seems a promising sedative adjunct as midazolam use is not supported by evidence.

## Introduction

Invasive mechanical ventilation has the potential to cause pain and distress.^1–3^ Over the past decade, despite a dramatic increase in the use of non-invasive ventilation in neonatal care, the majority of very premature infants continue to receive mechanical ventilation during parts of their NICU stay: 84% of infants born before 29 weeks in the US^4^ and 98% of infants born before 28 weeks in the UK^5^. Given the cumulative evidence of pain in infants^6^, and growing concerns regarding the potential long-term neurodevelopmental effects of pain and distress in early life^7^, the provision of appropriate and effective analgesia and sedation is paramount. However, there is ongoing controversy regarding the use of analgesics and sedatives in the context of mechanical ventilation in premature infants.^8,9^ As such, there is substantial variability, both within and between countries in the use of analgosedatives and their dosage in NICUs.^10,11^ This is likely due to a lack of knowledge regarding effective analgesic doses, the optimal degree of sedation, and uncertainty regarding associated acute adverse effects and long-term safety, including negative neurodevelopmental effects^12^.

A lack of consensus on the provision, type, and dosage of analgosedatives will inevitably result in some premature infants enduring untreated pain or others experiencing adverse effects from unnecessary treatment, with both outcomes having potential long-term consequences.^13^ Clinical decision-making requires a comprehensive understanding of the balance of benefits and risks of any potential treatment from the best available evidence. Therefore, the aim of this systematic scoping review was to identify which analgosedative drugs have been studied in ventilated premature infants and to objectively report their benefits and risks to guide future clinical management of this patient population and motivate further research.

## Methods

### Study design

The protocol for this review was developed in accordance with the PRISMA-P 2015 guidelines and checklist,^14^ and was publicly registered on 15th June 2022 on OSF, prior to data extraction (https://doi.org/10.17605/OSF.IO/YNHGS). This systematic scoping review aimed to assess the benefits and harms of pharmacological analgesics and sedatives used in premature neonates receiving invasive ventilation. We included all study designs from primary empirical research that were full peer-reviewed publications. A full list of eligibility criteria is provided in the Supplementary Information S.1. (Tables S1 and S2).

### Objectives

We conducted this scoping review to report the short and long-term beneficial and harmful outcomes associated with the use of analgesics and sedatives during invasive ventilation in premature infants. We sought to examine the results in the context of doses and open-label treatments and to identify gaps in our knowledge and research priorities.

### Search strategy

We searched five bibliographic databases to identify potentially relevant records on February 15th, 2022, with the assistance of an academic librarian: Embase (Embase.com), MEDLINE (Ovid Technologies, Inc), Web of Science Core Collection (Web of Knowledge), Cochrane Central Register of Controlled Trials (John Wiley & Sons), and the first 200 search results from Google Scholar (Publish or Perish). Additionally, we performed backward citation searching for all studies identified at the end of the screening process. The search was updated on February 12th, 2024. All search strategies are provided in full in the Supplementary Information S.2.

### Report selection

Search results were curated and de-duplicated in EndNote and uploaded to EPPI-Reviewer Web^15^ for review. Study selection was a two-stage process: screening on title and abstract followed by screening on full text. Screening was carried out in duplicate by two independent reviewers and disagreements settled by discussion between reviewers. Remaining disagreements were resolved by a third reviewer. To ensure standardised study selection process, an initial piloting stage was performed.

### Data extraction

Due to the high volume of reports eligible for data extraction (*n* = 80), the data extraction process was distributed among five reviewers (*n* = 15–16 reports each). Each reviewer’s data extraction results were validated by a second reviewer. Any disagreements were settled by discussion between reviewers. To ensure a standardized data extraction process, an initial piloting stage was performed. The standardized data extraction form listing all extracted data items is available via OSF (https://osf.io/xyjb4) and a summary of data items are listed in the Supplementary Information S.3.

## Results

### Summary of included studies

**Fig. 1:**
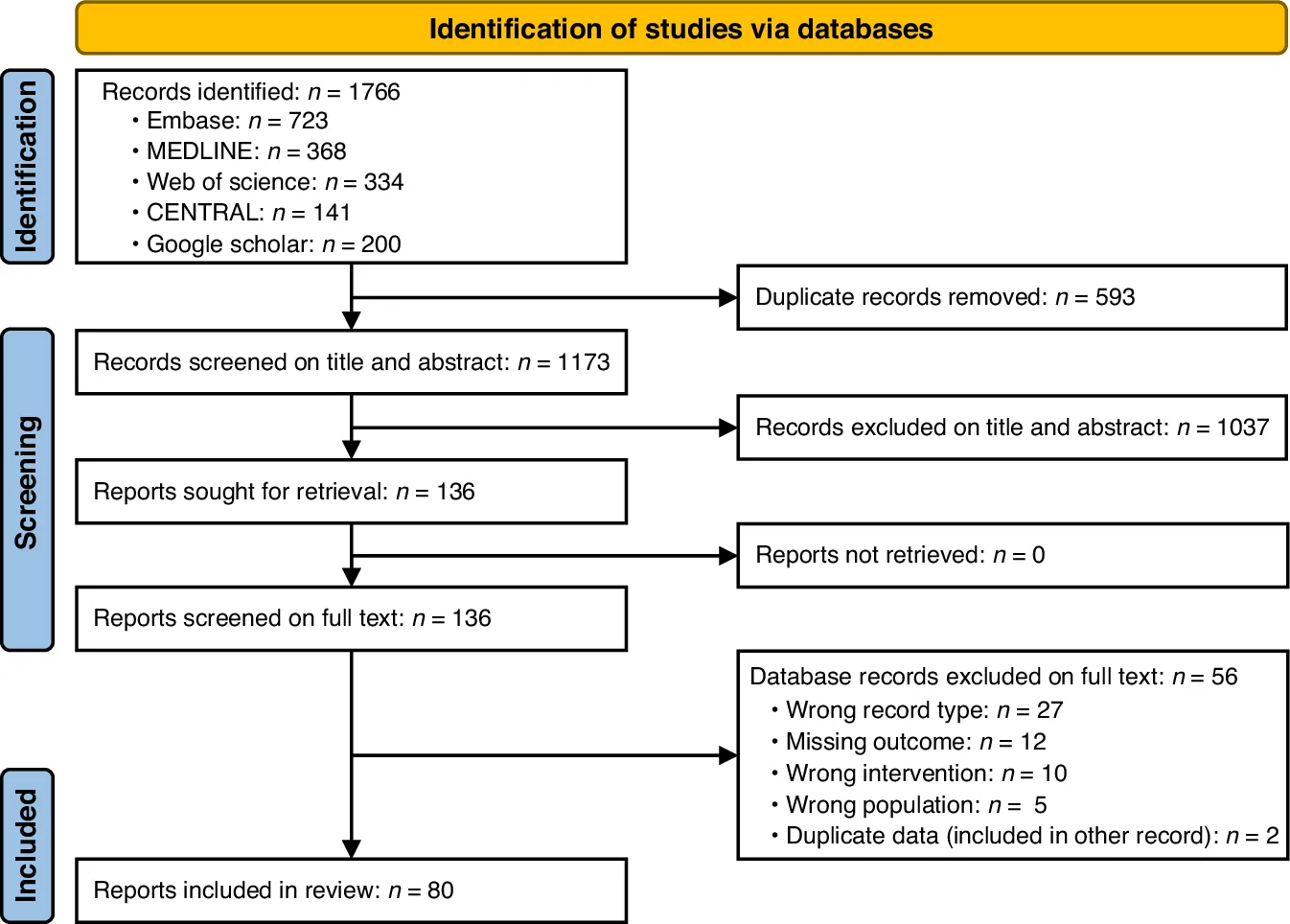
Prisma flow diagram. The flow chart illustrates the systematic process of study selection.

Our bibliographic database search yielded 1766 records, with 593 duplicates. 1173 records were screened on title and abstract. 136 reports were sought for retrieval. 82 relevant studies were identified via full text screening; 75 in English, others in Chinese,^16,17^ French,^18,19^ Portuguese^20^ and German,^21,22^ translated for data extraction. Some articles^21,23^ reported the same study, with considerable overlap of results. Therefore, only data from one^23^ were considered in the review. Similarly, the same patients and data were reported by two articles,^20,24^ therefore only data extracted from the later publication were included. The study selection process is outlined in Fig. 1. A summary of characteristics of the 80 studies included is presented in Table 1.

**Table 1:** Study characteristics.

Author	Year	Country	Study design	Primary	Centres	Sample	Age		Drug (n)	Comparator (n)	Primary aim (s)
				or secondary analysis		size	premature	Gestational age (weeks)			
**Studies of morphine**											
**Randomized controlled trials**											
Quinn^a^	1992	UK	open RCT	primary	1	95 (morphine: 29; pancuronium: 28; M + P: 38)	prem only	morphine: 29 [24–34]^f^; pancuronium: 28 [24–32]^f^; M + P 28 [24–33]^f^	Morphine	Pancuronium, Morphine + Pancuronium	Stress response
Quinn^a^	1993	UK	double-blind RCT	primary	1	41 (morphine 21; placebo: 20)	prem only	morphine: 28 (27–31)^g^; placebo 29 (27–31)^g^	Morphine	Placebo	Stress response
Dyke	1995	Australia	double-blind RCT	primary	1	26 (morphine: 12; placebo: 14)	prem only	morphine: 31 (29.25–33)^g^; placebo: 32 (29.75–34)^g^	Morphine	Placebo	Cardiovascular and respiratory outcomes
Wood	1998	UK	double-blind RCT	primary	1	88 (morphine: 44; diamorphine: 44)	prem only	morphine: 28 (26–30)^g^; diamorphine: 27 (26–29)^g^	Morphine	Diamorphine	Analgesia/sedation and safety
MacGregor^a^	1998	UK	follow-up of 2 RCTs	secondary	1	87 (morphine:57; control: 30)	prem only	morphine: 29 (27–31)^g^; non-morphine: 29 (27–30)^g^	Morphine	Pancuronium OR Placebo	Neurological outcome
Anand	1999	USA, Canada, Sweden, Scotland, Germany	pilot double-blind RCT	primary	9	67 (morphine: 24; midazolam: 22; placebo: 21)	prem only	midazolam 28.6 (2.5)^h^; morphine 29.2 (2.2)^h^; control 28.1 (2.2)^h^	Morphine	Placebo OR midazolam	Analgesia/sedation and safety
Simons^b^	2003	Netherlands	double-blind RCT	primary	2	150 (morphine 73; placebo: 77)	prem only	morphine: 29.1 (27.4–31.6)^g^; placebo: 29.2 (27.3–31.4)^g^	Morphine	Placebo	Analgesia/sedation
Anand^c^	2004	USA, France, Sweden, UK	double-blind RCT	primary	16	898 (morphine: 449; placebo: 449)	prem only	[23–32]^i^	Morphine	Placebo	Death and neurological outcome
Simons^b^	2005	Netherlands	double-blind RCT	secondary	2	126 (morphine 60; placebo 66)	prem only	morphine: 30.3 (27.5–32.1)^g^; placebo: 29.6 (28.4–32.1)^g^	Morphine	Placebo	Stress response
Hall^c^	2005	USA, France, Sweden, UK	double-blind RCT (ancillary)	secondary	16	898 (morphine: 449; placebo: 449)	prem only	[23–32]^i^	Morphine	Placebo	Cardiovascular outcome
Bhandari^102^^c^	2005	USA, France, Sweden, UK	double-blind RCT	secondary	16	898 (morphine: 449; placebo: 449)	prem only	morphine: 27.3 (2.3)^h^; placebo 27.4 (2.3)^h^	Morphine	Placebo	Respiratory outcome
Boyle^103^^c^	2006	UK	double-blind RCT (ancillary)	secondary	1	22 (morphine: 12; placebo: 10)	prem only	26 (23–31]^f^	Morphine	Placebo	Analgesia/sedation
Simons^b^	2006	Netherlands	double-blind RCT	secondary	2	144 (morphine: 71; placebo: 73)	prem only	morphine: 29 (27.4–31.8)^g^; placebo 29.1 (27.3–31.3)^g^	Morphine	Placebo	Cardiovascular outcome
Rao^104^^c^	2007	USA, France, Sweden, UK	follow-up study of RCT	secondary	16	572 (morphine: 275; placebo: 297)	prem only	27 [23–32]^f^	Morphine	Placebo	Neurological outcome
Cignacco	2008	Switzerland	double-blind RCT	primary	2	30 (morphine 16; placebo 14)	prem only	morphine: 28.17 (3)^h^; placebo: 28.08 (3.93)^h^	Morphine (bolus before suction)	Placebo	Analgesia/sedation
Menon^c^	2008	USA, France, Sweden, UK	double-blind RCT	secondary	16	898 (morphine: 449; control: 449)	prem only	227 [23–32]^f^	Morphine	Placebo	Gastrointestinal outcome
De Graaf^b^	2011	Netherlands	follow-up study of RCT	secondary	2	90 (morphine: 49; placebo: 41)	prem only	30.0 (27.5–31.6)^g^	Morphine	Placebo	Neurological outcome
Jiang	2012	China	double-blind RCT	primary	1	46 (morphine: 22; placebo: 24)	prem + term	≥32	Morphine	Placebo	Respiratory outcome
De Graaf^b^	2014	Netherlands	follow-up study of RCT	secondary	2	79 (morphine: 20; placebo: 20; control: 39)	prem + term	morphine: 29.8 (2.9)^h^; placebo: 30.2 (3.4)^h^	Morphine	Placebo	Stress response
Valkenburg^b^	2015	Netherlands	follow-up study of RCT	secondary	2	89 (morphine: 43; placebo: 46)	prem only	morphine: 30 (29–32)^g^; placebo: 31 (28-32)^g^	Morphine	Placebo	Neurological outcome
van den Bosch^b^	2015	Netherlands	follow-up study of RCT	secondary	1	19 (morphine: 15; no morphine: 4)	prem only	31.1 [26.1–36.3]^f^	Morphine	Control (no Morphine)	Neurological outcome
Välitalo^b^	2017	Netherlands	double-blind RCT	secondary	2	140 (morphine: 571; placebo: 569)	prem only	30.1 (3.5)^h^	Morphine	Placebo	Pharmacology
**Observational cohort studies**											
Hartley	1993	UK	prospective cohort	primary	1	17 with 2 dose regimen (9 and 8)	prem only	26–34]^i^, 29.6 (2.03)^h^	Morphine	n/a	Pharmacology
Miller^105^	1994	USA	prospective cohort	primary	1	9	prem only	[29-32]^i^	Morphine ( + pancuronium)	n/a	Respiratory outcome and safety
Sabatino	1996	Italy	prospective cohort	primary	1	30	prem only	29 (2)^h^, [27–31]^i^	Morphine	n/a	Cardiovascular outcome
Rutter^106^	2000	Australia	prospective cohort	primary	1	17	prem only	27.0 [24–32]^f^	Morphine	n/a	Cardiovascular outcome
Saarenmaa^d^	2000	Finland	prospective cohort	secondary	1	31	prem + term	30 (28–34)^g^	Morphine	n/a	Pharmacology
Anand^c^	2008	USA, France, Sweden, UK	prospective cohort	secondary	16	875	prem only	[23–32]^i^	Morphine	n/a	Pharmacology
Duong^107^	2020	France	retrospective cohort	primary	1	17	prem only	25.9 (24.6–26.9)^g^	Morphine (oral)	Morphine (intravenous)	Analgesia/sedation
**Observational case-control studies**											
Quinn	2000	UK	prospective case-control	secondary	1	40 (morphine 14; control 26)	prem only	morphine: 30 [24–34]^f^; no morphine 28 [24–35]^f^	Morphine	Control (no Morphine)	Respiratory outcome
Fleishman	2013	USA	retrospective case-control	primary	1	410 (morphine: 129; no morphine: 281)	prem only	no morphine: 26.9 (2)^h^; morphine: 26.4 (2)^h^	Morphine	Control (no Morphine)	
Fleishman	2015	USA	retrospective and prospective case-control	primary	1	134 (standard morphine: 52; non-standard morphine: 82)	prem only	Standard morphine: 26.6 (1.5)^h^; non-standard morphine 26.3 (1.3)^h^	Morphine (standardized)	Morphine (non-standardized)	Respiratory, gastrointestinal and neurological outcome
**Case reports**											
Barr^108^	1981	Australia	case report	n/a	1	1	premature	30	Morphine	n/a	Respiratory outcome
Musharaf^109^	2009	ND	case report	n/a	1	1	premature	25	Morphine	n/a	Renal effect
**Studies of fentanyl**											
**Randomized controlled trials**											
Orsini	1996	USA	double-blind RCT	primary	1	20 (fentanyl 11; placebo 9)	prem only	fentanyl: 31.6 (2.8)^h^; placebo: 29.9 (3.2)^h^	Fentanyl	Placebo	Neurological, respiratory, cardiovascular outcomes and stress response
Guinsburg	1998	Brazil and USA	double-blind RCT	primary	1	22 (fentanyl: 11; placebo: 11)	prem only	fentanyl: 31 (1)^h^, placebo: 30 (2)^h^	Fentanyl	Placebo	Cardiovascular outcome, analgesia/sedation and stress response
Lago	1998	Italy	open RCT	primary	1	53 (fentanyl: 27; placebo: 28)	prem only	fentanyl: 31 (2)^h^; control 31 (2)^h^	Fentanyl	Placebo	Analgesia/sedation, stress response, cardiovascular, respiratory, gastrointestinal and neurological outcomes
Saarenmaa^d^	1999	Finland	double-blind RCT	primary	1	163 (fentanyl: 83; morphine: 80)	prem + term	fentanyl 31.7(29.4–37)^g^; morphine: 31 (28.9–35.3)^g^	Fentanyl	Morphine	Analgesia/sedation, cardiovascular, respiratory outcomes, stress response and safety
Ancora^e^	2013	Italy	double-blind RCT	primary	5	131 (fentanyl: 64; placebo: 67)	prem only	fentanyl: 26 [22–32]^f^; control: 26 [22–31]^f^	Fentanyl	Placebo	Analgesia/sedation
Chen	2015	China	open RCT	primary	1	30 (fentanyl: 15; control: 15)	prem + term	[28–39]^i^; control: 34 (2.9)^h^; fentanyl 34.2 (3.9)^h^	Fentanyl	Control (no Fentanyl)	Cardiovascular outcome
Ancora^e^	2017	Italy	follow-up study of RCT	secondary	5	78 (fentanyl: 39; control: 39)	prem only	fentanyl: 25 [23–32]^f^; placebo: 26 [23–32]^f^	Fentanyl	Placebo	Neurological outcome
Abiramalatha	2019	India	open RCT	primary	1	100 (continous fentanyl: 53; bolus: 47)	prem + term	continuous: 36.5 (4.6)^h^; bolus: 35.4 (4.0)^h^	Fentanyl (intermittent boluses)	Fentanyl (continuous)	Pharmacology
Qiu	2019	China	double-blind RCT	primary	1	53 (fentanyl: 27; control: 26)	prem only	fentanyl: 31.1 (2.0)^h^; control: 30.3 (2.0)^h^	Fentanyl	Placebo	Analgesia/sedation, stress response and neurological outcome
**Observational case-control studies**											
Roth	1991	Germany	retrospective and prospective case-control	primary	1	40 (fentanyl: 20; control: 20)	prem + term	fentanyl: [26–40]^i^; control: [26–37]^i^	Fentanyl	Control (no Fentanyl)	Analgesia/sedation
Schmidt	2008	Germany	prospective case-control	primary	1	40 (fentanyl: 20; control: 20)	prem + term	fentanyl: 36.6 [28–42]^f^; control: 36.8 [30–41]^f^	Fentanyl (+continuous midazolam and pentobarbital or thiopental boluses)	Control (No fentanyl +continuous midazolam and pentobarbital or thiopental boluses)	Gastrointestinal outcome
Lammers	2014	USA	retrospective case-control	primary	1	147 (fentanyl high dose: 21; low/no dose: 126)	prem only	High dose: 27.0 (1.7)^h^, low/no dose: 29.2 (2.7)^h^	Fentanyl (high dose)	Fentanyl (low dose)	Neurological outcome
Abushanab	2019	Qatar	retrospective case-control	primary	1	126 (fentanyl: 63; morphine: 63)	prem + term	morphine prem: 28.77 (4.4)^h^; fentanyl prem: 30.49 (3.8)^h^ morphine term: 38.88 (1.1)^h^; fentanyl term: 39.6 (1.3)^h^	fentanyl	Morphine	Analgesia/sedation
**Case reports**											
Huet	1992	France	case report	n/a	1	1	premature	32	Fentanyl	n/a	Respiratory outcome
Lajarrige	1993	France	case report	n/a	1	1	premature	32	Fentanyl	n/a	Respiratory outcome
Pezzati	2001	Italy	case report	n/a	1	1	premature	32	Fentanyl	n/a	Gastrointestinal outcome
**Studies of other synthetic opioids**											
**Randomized controlled trials**											
Pokela	1994	Finland	double-blind RCT	primary	1	84 (meperidine: 42; placebo: 42)	prem + term	meperidine: 31.6 [25-40]^j^; placebo: 32.9 [24–41]^j^	Meperidine	Placebo	Cardiovascular and respiratory outcomes
Barker	1995	UK	double-blind RCT	primary	1	27 (diamorphine high dose: 14; low dose: 13)	prem + term	29 [24–42]^f^; low dose: 29 (27–30)^g^, high dose: 29 (27–32)^g^	Diamorphine	n/a	Analgesia, cardiovascular, respiratory and stress outcomes
Saarenmaa	1996	Finland	double-blind crossover RCT	primary	1	10 (alfentanil)	prem only	32 [29–36]^j^	Alfentanil	Placebo	Analgesia/sedation
Pereira e Silva	2008	Brazil	double-blind RCT	primary	1	40 (remifentanil: 20; morphine: 20)	prem only	remifentanil: 31.3 (1.5)^h^; morphine: 31.4 (1.7)^h^	Remifentanil	Morphine	Respiratory outcome
**Observational cohort studies**											
Marlow	1990	UK	prospective cohort	primary	1	22 (alfentanil)	prem only	30 [25–36]^f^	Alfentanil	n/a	Pharmacology
Elias-Jones^110^	1991	UK	prospective cohort	primary	1	34 (diamorphine)	prem + term	31.0 (4.0)^h^; [26–40]^i^	Diamorphine	n/a	Cardiovascular outcome
Pokela	1992	Finland	prospective cohort	primary	1	20 (alfentanil 19; placebo + alfentanil 1)	prem + term	36 [30–40]^f^	Alfentanil	n/a	Safety
Seguin	1994	USA	prospective cohort	primary	1	8 (sufentanil)	prem + term	37 [30–42]^f^	Sufentanil	n/a	Respiratory outcome and safety
Stoppa^111^	2004	Italy	prospective cohort	primary	1	18 (remifentanil)	prem + term	>32	Remifentanil	n/a	Analgesia/sedation
Giannantonio	2009	Italy	prospective cohort	primary	1	48 (remifentanil)	prem only	28.5 (2.5)^h^; [25–33]^i^	Remifentanil	n/a	Analgesia/sedation
**Observational case-control studies**											
Avenarius	2000	Germany	retrospective case-control	primary	1	38 (sufentanil: 19; control: 19)	prem + term	sufentanil: 32.6 (2.6)^h^; control: 32.3 (2.6)^h^	Sufentanil	Phenobarbital	Cardiovascular, respiratory and gastrointestinal outcomes
**Case reports**											
Pereira e silva	2005	Brazil	case report	n/a	1	1	premature	34	Remifentanil	n/a	Analgesia/sedation, cardiovascular and respiratory outcomes
**Studies of sedatives**											
**Randomized controlled trial**											
Jacqz-Aigrain	1994	France	double-blind RCT	primary	1	46 (midazolam: 24; placebo: 22)	prem only	midazolam: 32.1 (2.8)^h^; placebo: 32.8 (2.6)^h^	Midazolam	Placebo	Analgesia/sedation, cardiovascular, respiratory and neurological outcomes
Arya	2001	India	double-blind RCT	primary	1	33 (midazolam + morphine: 17; placebo + morphine: 16)	prem only	midazolam: 31.5 (2.4)^h^; placebo: 32.3 (2.2)^h^	Midazolam + Morphine	Placebo + Morphine	Analgesia/sedation
van Alfen- van der Velden	2006	Netherlands	open RCT	primary	1	21 (midazolam: 11; morphine: 10)	prem only	midazolam: [26.6–33.0]^i^; morphine: [26.4–33.3]^i^	Midazolam	Morphine	Cardiovascular outcome
**Observational cohort studies**											
Jorch	1990	Germany	prospective cohort	primary	1	11 (diazepam)	prem only	27 [25–30]^f^	Diazepam	n/a	Cardiovascular outcome
Jacqz-Aigrain	1992	France	prospective cohort	primary	1	15 (midazolam)	prem + term	32.8 (3.3)^h^; [29–41]^i^	Midazolam	n/a	Pharmacology
Harte	1997	Australia	prospective cohort	primary	1	10 (midazolam)	prem only	27.9 [25–30]^jf^	Midazolam (single dose)	n/a	Cardiovascular outcome
Treluyer	2005	France	prospective cohort	primary	1	23 (midazolam)	prem + term	>33	Midazolam	n/a	Analgesia/sedation
Chrysostomou	2014	USA	prospective cohort	primary	11	42 (dexmedetomidine 3 doses, *n* = 14 per group)	prem + term	prem: 31.8 (2.4)^h^; term: 38.7 (2.0)^h^	Dexmedetomidine	n/a	Analgesia/sedation
**Observational case-control studies**											
Bell	1993	Denmark	retrospective case-control	primary	2	77 (phenobarbitone: 37; morphine:18; control: 22)	prem only	29.0 (2.0)^h^; [25–32]^i^	Phenobarbitone	Morphine (boluses) OR Control	Neurological outcome
O’Mara	2012	USA	retrospective case-control	primary	1	48 (dexmedetomidine: 24; fentanyl: 24)	prem only	fentanyl: 24.9 (1.6)^h^; dexmedetomidine: 25.5 (1.7)^h^	Dexmedetomidine	Fentanyl	Analgesia/sedation and safety
Abushanab	2021	Qatar	retrospective case-control	primary	1	104 (morphine + midazolam: 52; morphine: 52)	prem + term	prem: midazolam: 26.5 (2.9)^h^; no midazolam: 28.2 (4.5)^h^term: midazolam: 39.3 (1.1)^h^; no midazolam: 38.6 (1.1)^h^	Midazolam	Morphine + Midazolam	Analgesia/sedation
**Case reports**											
Reiter^112^	1993	USA	case report	n/a	1	1	premature	33	Lorazepam	n/a	Safety
O’Mara	2009	USA	case report	n/a	1	1	premature	24	Dexmedetomidine	n/a	Analgesia/sedation
**Studies of mixed narcotics and/or sedatives**											
**Observational case-control studies**											
Kahn	1998	USA	prospective case-control	secondary	6	1018 (narcotics: 196, no narcotics: 822)	prem only	narcotics: 27.5 (2.6)^h^; no narcotics: 28.5 (2.8)^h^	Narcotics	No narcotics	Respiratory, cardiovascular, neurological outcomes
Avila-alvarez^113^	2015	Spain	prospective case-control	primary	30	202 (analgesics or sedatives: 158; none: 44)	prem + term	33.9 (29.1–38)^g^	Analgesics or sedatives	None	No outcome
Toye	2019	Canada	retrospective case-control	primary	30	2672 (none: 1805; sedatives: 101; narcotics: 467; both:299)	prem only	No sedatives or narcotics: 28.8 (2.7)^h^; sedatives: 27.0 (2.4)^h^; narcotics: 27.3 (3.0)^h^; both: 27.2 (3.2)^h^	Narcotics/Sedatives/Narcotics + sedatives	No sedatives or narcotics	Death, respiratory and neurological outcomes
De Tristan	2021	France	prospective case-control	secondary	402	922 (450 narcotics and/or midazolam and 472 no narcotics or midazolam)	prem only	[23–31]^i^	Narcotics and/or midazolam	No narcotics or midazolam	Death and neurological outcomes
Szatkowski	2023	UK	retrospective case-control	primary	ND	24815 (narcotics: 20561; no narcotics: 4254)	prem only	narcotics: 26 (25–28)^g^; no narcotics: 27 (26–29)^g^	Narcotics	No narcotics	Death, neurological and respiratory outcomes

*RCT* randomized control trial.

^a,b,c,d,e^Refer to related studies.

^f^Median [range].

^g^Median (interquartile range).

^h^Mean (SD).

^i^[range].

^j^Mean [range].

Studies were published between 1981 and 2023. Only 10% (*n* = 8) were conducted in the last 5 years. Most studies reported research conducted in Europe (*n* = 46; 57%). Others were based in North America (*n* = 12; 15%), Asia (*n* = 7; 9%), Australia (*n* = 4; 5%), and South America (*n* = 2; 3%). 10% of studies were international (*n* = 8); one study did not disclose a location.^25^ Study designs were largely randomized controlled trials (RCTs), including 25 double-blind (31%), 5 open (6%), 1 pilot double-blind (1%), and 7 follow-up studies of RCTs (9%). The rest comprised of 19 cohort studies (24%), 15 case-control studies (19%), and eight case reports (10%). Most studies were primary (*n* = 52; 65%), monocentric (*n* = 56; 70%), and included only premature infants (*n* = 59; 74%). The most common study aims were assessment of analgesia and/or sedation (*n* = 25). Other aims included respiratory (*n* = 21), cardiovascular (*n* = 19), neurological (*n* = 19), stress hormone (*n* = 10), safety (*n* = 8), pharmacological (*n* = 7), gastrointestinal (*n* = 7), death (*n* = 3), and renal outcomes (*n* = 1). Many studies included a placebo group for comparison (29 of 55 studies that included a comparator group).

The most frequently studied drugs were morphine (*n* = 34; 42%) and fentanyl (*n* = 16; 20%). Other studies investigated the effects of alternative synthetic opioids (*n* = 12; 15%) such as remifentanil, alfentanil, sufentanil, diamorphine, meperidine, or sedative agents (*n* = 13; 16%) including dexmedetomidine, lorazepam, midazolam, diazepam and phenobarbitone. Five studies included a mixture of narcotics and/or sedatives (*n* = 5; 6%). Sample sizes ranged from single case report studies to large observational case-control studies with 2672 patients,^26^ and included infants as young as 22 weeks’ gestation^27^ through to term.

We have classified the studies by drug, reporting the results within the categories of morphine, fentanyl, other synthetic opioids, sedatives, and mixed studies of narcotics and/or sedatives. For each of these categories, we have summarized the significant benefits and risks reported (Tables 2–6).

**Table 2:** Studies of morphine.

Author (year)	Sample size	Comparator	Morphine dose			Analgesia			Sedation	Respiratory effects						Cardiovascular effects				Neurological effects		Gastrointestinal effect		Stress response						
	total (morphine)		Loading (µg/kg)	Continuous (µg/kg/h)	open label yes/no	Validated pain score	Reliability assessment	Analgesic efficacy		pO2/SpO2	Respiratory rate	Ventilation parameters	Duration mechanical ventilation	Bronchopulmonary dysplasia	Pneumothorax	Heart Rate	Blood pressure	Patent ductus arteriosus	Vasoactive treatment	IVH/PVL	Other neurological	Time to feed	Necrotizing enterocolitis		Sepsis	Renal effect urinary retention	Withdrawal	Adverse events	Mortality	Illness severity included in analysis
***RCT studies***																														
***Morphine vs placebo***																														
Quinn^32a^	41 (morphine 21)	placebo: 20	100	25	no	Yes (scale not specified)	-	no difference at 0 or 24 h	-	-	-	5% more FiO2 in 6 h (*p* = 0.07)	no difference	-	no difference	no difference (in 6 h)	no sig difference (in 6 h)	no difference	-	no difference IVH	-	-	-	decrease adrenaline in 24 h‡; no difference noradrenaline	-	-	-	None	no difference	Lung disease severity and cardiovascular status balanced at baseline
Dyke^30^	26 (morphine: 12)	placebo: 14	100	10	no	-	-	-	-	no difference between groups in response to ET suction	lower with morphine over 48 h†	higher ventilator synchrony with morphine over 48 h‡	no difference MV, shorter oxygen therapy (*p* = 0.046)	no difference	no difference	lower with morphine over 48 h†	no difference over 48 h	-	-	no difference IVH	-	-	-	-	-	-	-	-	-	Lung disease severity balanced at baseline
Simons^38b^	150 (morphine 73)	placebo: 77	100	10	yes	VAS, NIPS, and PIPP	Yes	no difference 30 min after start infusion or before/during/30 min post-ET suctions)	-	-	-	-	no difference	no difference	-	-	-	no difference	-	less IVH (all grades), no difference in poor neurological outcome (IVH/PVL/death)	-	-	no difference	-	-	-	-	1 overdose	no difference (5% vs 9%; no stats)	CRIB score in logistic regression
Anand^39c^	898 (morphine: 449)	placebo: 449	100	10–30	yes	PIPP	-	lower PIPP to ET suction at 24 h vs placebo†, not 72 h	-	-	*lower 24 h after start‡*	-	longer MV†	no difference	-	lower at 72 h‡	lower after loading dose and within 24 h (no difference >24 h)	-	-	overall no difference IVH/PVL/death morphine vs placebo; more severe IVH in 27–29 weeks†. Infants without open label: more IVH/PVL/death† and severe IVH† with morphine. Infants with open label: more severe IVH in open label morphine group‡	-	longer time to full enteral feeding †, no difference duration IV nutrition	-	-	-	no difference	-	-	no difference	CRIB score in logistic regression
*Bhandari*^c^	*898 (morphine: 449)*	*placebo: 449*	*100*	*10–30*	*yes*	*-*	*-*	*-*	*-*	*-*	*-*	*-*	*longer MV†, no difference nCPAP or O2*	*no difference*	*no difference*	*-*	*-*	*no difference*	*-*	*-*	*-*	*-*	*-*	*-*	*no difference*	*-*	*-*	*-*	*no difference*	*CRIB score and illness factors in logistic regression*
*Hall*^47c^	*898 (morphine: 449)*	*placebo: 449*	*100*	*10–30*	*yes*	*-*	*-*	*-*	*-*	*-*	*-*	*-*	*-*	*-*	*-*	*-*	more hypotension during loading‡ and in 24 h of infusion‡; highest incidence in 23–26 week GA	*-*	*-*	morphine not associated with severe IVH or any IVH.	*-*	*-*	*-*	*-*	*-*	*-*	*-*	*-*	morphine not associated	*CRIB score and illness factors in logistic regression*
*Simons*^48^^b^	*126 (morphine 60)*	*placebo 66*	*100*	*10*	*yes*	*-*	*-*	*-*	*-*	*-*	*-*	*-*	*no difference*	*-*	*-*	*-*	*-*	*-*	*-*	*IVH 18% vs 38% (no stats) poor neurological outcome 5% vs 15% (no stats)*	*-*	*-*	*-*	lower noradrenaline over 96 h†*, no difference adrenaline*	*-*	*-*	*-*	*-*	*-*	*CRIB score balanced at baseline*
*Boyle*^c^	*22 (morphine: 12)*	*placebo: 10*	*100*	*10–30*	*no*	*-*	*-*	*-*	*-*	*-*	*-*	*Poor ventilator synchrony associated with placebo*	*-*	*-*	*-*	*-*	*-*	*-*	*-*	*-*	*-*	*-*	*-*	*-*	*-*	*-*	*-*	*-*	*-*	*-*
*Simons*^48^^b^	*144 (morphine: 71)*	*placebo: 73*	*100*	*10*	*yes*	*-*	*-*	*-*	*-*	*-*	*-*	*-*	*-*	*-*	*-*	*-*	*more hypotension in 48 h of infusion‡ (70% vs 47%), no difference MABP or BP variability*	*-*	*no difference*	*no increase in IVH in hypotensive patients*	*-*	*-*	*-*	*-*	*-*	*-*	*-*	*-*	*-*	*CRIB score in logistic regression*
*Rao*^c^	*572 (morphine: 275)*	*placebo: 297*	*100*	*10–30*	*yes*	*-*	*-*	*-*	*-*	*-*	*-*	*-*	*-*	*-*	*-*	*-*	*-*	*-*	*-*	*-*	*Neurobehavioural Assessment of Preterm Infant (NAPI) score at 36 weeks: higher popliteal angle score†*	*-*	*-*	*-*	*-*	*-*	*-*	*-*	*-*	*CRIB score and Neonatal Medical Index in logistic regression*
Cignacco^40^	30 (morphine 16)	placebo 14	100 then 50	-	-	BPSN, VAS, PIPP	-	no difference after bolus, during suction, or after comfort measures	-	-	-	-	no difference	-	-	-	-	-	-	-	no difference head circumference at discharge	-	-	-	-	-	-	-	-	-
*Menon*^50^^c^	*898 (morphine: 449)*	*placebo: 449*	*100*	*10–30*	*yes*	*-*	*-*	*-*	*-*	*-*	*-*	*-*	*-*	*-*	*-*	*-*	*-*	*-*	*-*	*-*	*-*	*later starting feeding and reaching full feeds‡*	*no difference*	*-*	*-*	*-*	*-*	*-*	*-*	*CRIB score in logistic regression*
*DeGraaf*^114^^b*, X*^	*90 (morphine: 49)*	*placebo: 41*	*100*	*10*	*yes*	*-*	*-*	*-*	*-*	*-*	*-*	*-*	*-*	*-*	*-*	*-*	*-*	*-*	*-*	*-*	*no difference IQ, visual-motor integration, behavior, chronic pain or quality of life at 5 years*	*-*	*-*	*-*	*-*	*-*	*-*	*-*	*-*	*CRIB score (propensity score) in logistic regression*
Jiang^16^	46 (morphine: 22)	placebo: 24	100	10	-	N-PASS and COMFORT	-	lower scores vs placebo at 2 h† and 12 h‡	lower scores (2 h and 12 h)	-	-	no difference (in 48 h)	no difference	-	no difference	lower at 24–48 h‡	no difference	no difference	-	no difference (IVH and PVL)	-	-	no difference		-	-	-	no difference	-	-
*DeGraaf*^37^^b*, X*^	*79 (morphine: 20)*	*placebo: 20; term born control: 39*	*100*	*10*	*yes*	*-*	*-*	*-*	*-*	*-*	*-*	*-*	*-*	*-*	*-*	*-*	*-*	*-*	*-*	*-*	*-*	*-*	*-*	*no difference in salivary cortisol at 5 years*	*-*	*-*	*-*	*-*	*-*	*CRIB score and other characteristics balanced at baseline*
*Valkenburg*^115^^b, *X*^	*89 (morphine: 43)*	*placebo: 46*	*100*	*10*	*yes*	*-*	*-*	*-*	*-*	*-*	*-*	*-*	*-*	*-*	*-*	*-*	*-*	*-*	*-*	*-*	*no difference in thermal detection, pain threshold, incidence of chronic pain, neurological functioning (IQ) at 8–9 years*	*-*	*-*	*-*	*-*	*-*	*-*	*-*	*-*	*CRIB score (propensity score) in logistic regression*
*Van den Bosch*^36^^b*, X*^	*19 (morphine: 15)*	*placebo 4*	*100*	*10*	*yes*	*-*	*-*	*-*	*-*	*-*	*-*	*-*	*-*	*-*	*-*	*-*	*-*	*-*	*-*	*-*	*opioid exposure associated with brain volume, no association with neuropsychological functioning or thermal sensitivity at 8-15yrs*	*-*	*-*	*-*	*-*	*-*	*-*	*-*	*-*	*-*
*Välitalo*^41^^b^	*140 (morphine: 571)*	*placebo: 569*	*100*	*10*	*yes*	*VAS, NIPS, PIPP*	*Yes*	*Non clinically relevant analgesic effect during/after ET suction*	*-*	*-*	*-*	*-*	*-*	*-*	*-*	*-*	*-*	*-*	*-*	*-*	*-*	*-*	*-*	*-*	*-*	*-*	*-*	*-*	*-*	*-*
***Morphine vs placebo or other drug***																														
*MacGregor*^35^^a*, X*^	*87 (morphine: 57)*	control (placebo or pancuronium): 30	*None or 100*	*25–100*	*no*	*-*	*-*	*-*	*-*	*-*	*-*	*-*	*-*	*-*	*-*	*-*	*-*	*-*	*-*	*no difference*	*disability 13% versus 8%, no difference IQ, behavior or motor function at 5–6 years*	*-*	*-*	*-*	*-*	*-*	*-*	*-*	*no difference*	*-*
Anand^34^^f^	67 (morphine: 24)	midazolam: 22; placebo: 21	100	10–30	yes	PIPP	-	lower PIPP to ET suction vs placebo‡ (timepoint not specified)	COMFORT score increased at 12h after stopping morphine‡	-	-	-	no difference	-	no difference	-	-	-	-	fewer poor neurological outcomes (IVH/PVL/death)	no difference in NAPI scores at 36 wks	no difference	-	-	-	-	-	-	no difference	CRIB score balanced between groups
***Morphine vs other drug(s)***																														
Quinn^29^^a^	95 (morphine: 29)	pancuronium: 28; morphine and pancuronium: 38	-	50–100	no	-	-	-	-	-	-	no difference PIP and FiO2 at 6 h	no difference	-	no difference	no difference at 6 h	increased from baseline at 6 h in M + P group	no difference		no difference (no stats)	-		-	morphine decreased noradrenaline at 24 h†; no change in adrenaline	-	-	-	-	no difference	-
Wood^44^^g^	88 (morphine: 44)	diamorphine: 44	200	25	no	-	-	-	quicker sedation with diamorphine, no difference at 24 h	-	-	no difference (oxygen at 28 days)	no difference		no difference		reduced BP after morphine loading†, no diff in BP variability between groups	no difference	no difference (45% vs 32%)	no difference IVH (34% vs 52%)	-	-	-	morphine reduced adrenaline‡ and noradrenaline‡ at 24 h	-	-	-	-	no difference	Some cardiorespiratory indices balanced at baseline
Saarenmaa^42^^d,^^h^	163 (morphine: 80)	fentanyl: 83	140	20	yes	Adapted NIPS	-	no difference between groups in change in pain scores to ET suction at 2–12, 12–24, and 24–48 h	-	-	-	-	-	-	-	no difference in HR at 2 or 24 h for M or F	no difference (no data)	-	no difference	no difference	-	more decreased intestinal motility‡ (M 47% vs F 23%) no difference beginning enteral feeding	no difference	morphine reduced adrenaline‡ and noradrenaline† at 24 h; no diff in noradrenaline, adrenaline and B-endorphin decrease between groups	no difference	no difference	none	no difference	no difference	Illnesses balanced at baseline
van Alfen-van der Velden^49^^f^	21 (morphine: 10)	midazolam: 11	50	10	no	-	-	-	-	lower SaO2 and tcPO2 after infusion started (*n* = 6); increased SaO2 over 120 min after	-	increased fiO2 needed in 2 patients	-	-	-	small significant decrease in HR over 120 min	no difference over 120 min	-	-	-	Increase in cerebral blood volume† (maximal 120 min after start infusion)	-	-	-	-	-	-	-	-	-
Pereira e Silva^43^^g^	40 (morphine: 20)	remifentanil: 20	150	10	no	NIPS		no difference between groups during infusion or in 6 h post-extubation	no difference COMFORT scores between groups during infusion or in 6 h post-extubation	no difference between groups during 6 h post-extubation	-	lower mean airway pressure with morphine 5, 10, 15 min† post-start infusion	longer time to extubation†	-	-	-	no difference hypotension (no stats)	-	no difference (*n* = 6 vs *n* = 3; no stats)	-	no difference neurological evaluations at discharge	no difference	-	-	-	-	-	-	-	RDS severity balanced at baseline
**Observational cohort study**																														
Hartley^28^	17	-	100 or 200	12.5 or 50	-	-	-	-	-	-	-	-	-	-	-	-	lower in high dose (not significant)	-	-	-	no seizures, n=2 hypertonia in high dose	-	-	-	-	-	-	-	-	NA
Miller	9 (+pancuronium)	-	10–50	-	-	-	-	-	-	higher PaCO2 within 20 min infusion†	-	lower FRC within 20 min infusion†; lower tidal volume and minute volume (not significant)	-	-	-	-	-	-	-	-	-	-	-	-	-	-	-	-	-	NA
Sabatino^116^	30	-	100	25	-	-	-	-	-	no difference in tcPO2 and tcPCO2 at 15, 30, 60, and 120 min of infusion	-	-	-	-	-	no difference at 15, 30, 60, and 120 min of infusion	no change cardiac output or MABP at 15, 30, 60, and 120 min of infusion	-	*n* = 1	IVH *n* = 3; *n* = 2 PVL	no change in cerebral doppler flow during 2 h infusion	-	-	-	-	-	-	-	-	NA
Rutter	17	-	100	-	-	-	-	-		-	-	-	-	-	-	lower at 10† and 60‡ min after bolus	no difference in BP at 10 and 60 min after bolus (or right ventricular output, superior blood caval flow)	no difference in duct size at 10 and 60 min after bolus	-	-	-	-	-	-	-	-	-	-	-	NA
*Saarenmaa*^51d^	*31*	*-*	*140*	*20*	*yes*	*Adapted NIPS*	*-*	*no correlation between score for ET suctions and concentration at 24–48 h*	*-*	*-*	*-*	*-*	*-*	*-*	*-*	*-*	*-*	*-*	*-*	*-*	*-*	*higher concentrations when reduced intestinal motility†*	*-*	*-*	*-*	*-*	*-*	*-*	*-*	NA
*Anand*^88c^	*875*	*-*	*100*	*10–30*	*yes*	*PIPP*	*-*	*no correlation of concentration with PIPP at ET suction*	*-*	*-*	*-*	*-*	*-*	*-*	*-*	*no correlation of concentration with HR post-suction*	*-*	*-*	*-*	*-*	*-*	*-*	*-*	*-*	*-*	*-*	*-*	*-*	*-*	NA
Duong	17	-	-	-	-	COMFORTneo	-	no change in scores performed 6 hourly for 48 h after IV to oral switch	-	-	-	no change in MAP or FiO2 between 48 h of IV and 48 h of oral switch	-	-	-	-	-	-	-	-	-	-	-	-	-	-	65% (11/17) withdrawal symptoms 3–26 days after oral switch (none in 48 h)	-	-	NA
**Observational case-control study**																														
***Morphine vs no morphine***																														
*Quinn*^32^	*40 (morphine 14)*	*no morphine 26*	*50–100*	*5–15*	*-*	*-*	*-*	*-*	*-*	*no difference A/a O2 ratios and PCO2 at 1,2, and 12 h*	*-*	greater reduction in triggered breath rate at 12 h with morphine (*p* = 0.01)	*-*	*-*		*-*	*non-clinically significant reduction MABP over 12 h*	*-*	*-*	*-*	*-*	*-*	*-*	*-*	*-*	*-*	*-*	*-*	*-*	Some cardiorespiratory indices balanced at baseline
Fleishman^46^	410 (morphine: 129)	no morphine: 281	-	-	-	-	-	-	-	-	-	-	longer MV‡	higher discharge rate on home O2‡	-	-	-	increased PDA ligation‡	-	more mod-severe IVH‡	-	longer time parenteral nutrition‡	no difference	-	no difference	-	-	-	higher‡ (morphine 20.9% vs 7.5%)	NA
Fleishman^31^	134 (standard morphine: 52)	non standard morphine (pre-emptive sedation): 82	25–50	5–10	yes	BIIP	-	-	-	-	-	-	more days MV with non-standard morphine‡	no difference in home O2	-	-	-	no difference	-	no difference	-	-	no difference	-	no difference late-onset sepsis	-	-	-	greater mortality with standard morphine† (20% vs 7%) Note palliative patients in group	NA
***Morphine vs other drug***																														
Bell^45^^f^	37 (morphine: 18)	phenobarbitone: 37	100–200	-	-	-	-	-	-	-	-	-	-	-	-	-	-	-	-	IVH 33% versus 5% (phenobarbitone) versus 0% (no sedation)	morphine increased burst interval (BI) on aEEG for 6 h (*p* < 0.01). No difference in BI between morphine and phenobarbitone.	-	-	-	-	-	-	-	33% versus 16% (phenobarbitone) versus 0% (no sedation)	-
Abushanab^72^^h^	126 (morphine: 63)	fentanyl: 63	100–200	15–30	-	PIPP	-	More successful pain relief (PIPP ≤7) (after infusion start, timing unclear)	-	no difference in desaturations (no stats or timepoint)	-	-	-	-	-	no difference (no data or timeframe)	-	-	-	-	-	-	-	-	-	-	2% versus 0% (no stats)	-	17% vs 2% (no statistics)	baseline characteristics included in multivariate analysis
**Case reports**																														
Barr	1	-	135	-	no	-	-	-	-	large fluctuations in tcPO2 within 3 h start of morphine; no change tcPCO2	-	-	-	-	-	-	-	-	-	-	-	-	-	-	-	-	-	suspected pulmonary hypertension	-	NA
Musharaf^25^	1	-	-	20	no	-	-	-	-	-	-	-	-	-	-	-	-	-	-	-	-	-	-	-	-	urinary retention, hydronephrosis, acute renal failure within 3 days of starting morphine (resolution after catheter)	-	-	-	NA

^*X*^indicates - outcomes assessed beyond neonatal period..

*Secondary studies marked in italics.*

*NA* not applicable.

†Indicates *p* < 0.05.

‡Indicates *p* < 0.01.

^a,b,c,d,e^Refer to related studies.

^f^Also in sedatives table.

^g^Also in synthetic opioids table.

^h^Also in fentanyl table.

**Table 3:** Studies of fentanyl.

Author (year)	Sample size total (fentanyl)	Comparator	Fentanyl dose			Analgesia			Sedation	Respiratory effects						Cardiovascular effects				Neurological effects		Gastrointestinal effect		Stress response						
			Loading (µg/kg)	Continuous (µg/kg/h)	open label yes/no	Validated pain score	Reliability assessment	Analgesic efficacy		Respiratory effects pO2/SpO2	Respiratory rate	Ventilation parameters	Duration Mechanical ventilation	Bronchopulmonary dysplasia	Pneumothorax	Heart Rate	Blood pressure	Patent ductus arteriosus	Vasoactive treatment	Intraventricular hemorrhage/periventricular leukomalacia	Other neurological	Time to feed	Necrotizing enterocolitis		Sepsis	Renal effect urinary retention	Withdrawal	Adverse events	Mortality	Illness severity included in analysis
***RCT studies***																														
***Fentanyl vs placebo***																														
Orsini^54^	20 (fentanyl: 11)	placebo: 9	5	2 for 72 h; 1 for 24 h; 0.5 for 24 h	no	No (Behavioral State score)	-	↓ non-validated score from 16 to 48 h of treatment initiation† (assessment from day 0 to day 5)	-	-	-	↑ mean airway pressure day 3‡; ↑PIP days 3 and 4‡, ↑PEEP days 2, 3, 4‡	slower weaning	no diff	no diff	↓ days 0–5‡	no diff	no diff	none	no diff IVH	-			no diff cortisol; ↓ 11-deoxycortisol days 3, 4, 5‡	no diff					-
Guinsburg^24^	22 (fentanyl: 11)	placebo: 11	3	-	no	NFCS + (Postoperative comfort scale)	-	no diff at 30 and 60 min of administration	-	no diff at 30 and 60 min of administration	-	no diff at 30 and 60 min of administration	-		-	↓ max and min HR at 30 and 60 min‡	no diff at 30 and 60 min of administration	-	-		-			No diff cortisol, lactate, glucose; increased GH after fentanyl†						-
Lago^53^	53 (fentanyl: 27)	placebo: 26	-	0.5–2; mean (SE) 1.1 (0.08)	no	No (behavioral sedation score adapted from Hartwig)	-		↓ non-validated score at 24, 48 and 72 h†	no diff (no specified timeframe)	-	no diff (no specified timeframe)	no diff (hospital stay)	no diff	no diff	-	-	no diff	-	no diff (grade III-IV IVH or PVL)	-	no diff	no diff				no diff	no diff	no diff	CRIB score balanced at baseline
*Ancora*^27^^a^	131 (fentanyl: 64)	placebo: 67	1	1	yes	EDIN and PIPP	-	↓ PIPP on days 1–3 but not 4–7†; no diff EDIN (EDIN > 6: less in fentanyl days 1–7)	-	-	-	↑ MAP on days 5, 7†	no diff during hospitalization	no diff	-		no diff on days 1–6 (↑ BP day 7)	no diff	21% vs 25% (no specified timeframe)	no diff IVH/PVL	-	no diff	no diff	-	-	no diff	-	-	no diff	CRIB score balanced at baseline
Chen^55^ ^X^	30 (fentanyl: 15)	placebo: 15	2	2	no	PIPP	-	↓ PIPP 30 min, 2 h and 4 h after administration†	-	no diff 30 min, 2 h and 4 h after administration	↓RR 30 min, 2 h and 4 h after administration†	-	-	-	-	↓ (details unclear)	no diff 30 min, 2 h and 4 h after administration	-	-	-	No diff in MDI PDI at 3, 6, 9 and 12 months of age									-
*Ancora*^60^^a,^ ^X^	*78 (fentanyl: 39)*	*placebo: 39*	*1*	*1*	*yes*	*-*	*-*	*-*	*-*	*-*	*-*	*-*	no difference *during hospitalization*	*-*	*-*	*-*	*-*	*-*	*-*	*no diff severe brain damage at discharge or Developmental Quotient at 24 months. Reduced eye-hand coordination at 24 months‡*	*-*	*-*	*-*	*-*	*-*	*-*	*-*	*-*	*-*	Adjusted on CRIB score and sex
Qiu^52^	53 (fentanyl: 27)	placebo: 26	1	1 → 0.5	*no*	PIPP	-	↓ PIPP at 2, 12, 24, 48 h compared to placebo†	-	*-*	*-*	*-*	*-*	*-*	*-*	*-*	*-*	*-*	*-*	*-*	No diff in cerebrovascular parameters; fentanyl: reduced neuron-specific enolase†. increased CFM score†	*-*	*-*	*-*	*-*	*-*	*-*	*-*	*-*	-
***Fentanyl vs other drugs***																														
Saarenmaa^65^	163 (fentanyl: 83)	morphine: 80	10.5	1.5	no	No (adapted from NIPS)	-	no diff non-validated score (2, 12, 24 and48h)	-	-	-	-	no diff (no specified timeframe)		-	no diff (no specified timeframe)	no diff during opioid infusion	-	76% vs 84%	no diff grade III/IV (fentanyl 8%; morphine 5%)	-	↓ ≤1500 g; >1500 g no diff	no diff	no diff adre/noradrenaline	no diff	no diff	no diff	1+ AE: 72 % vs 68%	no diff	Illnesses balanced at baseline
**Fentanyl vs other**																														
Abiramalatha^61^	100 (continuous fentanyl: 53)	boluses: 47	1; boluses: 1 every 4 h	1 (continuous group)	no	NIPS and N-PASS	-	median NIPS (1-3), median NPASS (0–3) suggest no/mild pain during 48h	Low N-PASS and NIPS in both groups at all time points during 48h (median score indicates deep sedation)	-	-	no diff during opioid treatment	no diff during opioid treatment	-	-	-	no diff hypotensive schock during opioid treatment	-	83% vs 83% during opioid treatment			-	-	-	-	no diff		no diff	continuous 13%, boluses 19%	Illnesses balanced at baseline
**Observational cohort study**																														
***Fentanyl vs no fentanyl***																														
Roth^23^^b^	40 (fentanyl: 20)	control: 20	5–12.5	0.5–2.0	*-*	no	-	*-*	Satisfactory non validated scale ↓adjunctive sedation during invasive ventilation (no timeframe)	*-*	*-*	*-*	*-*	*-*	*-*	no difference (no timeframe)	no difference on days 1, 2, and 3	*-*	↓ catecholamine use during invasive ventilation (no specified timeframe)	*-*	*-*	Delayed first mecomium	*-*	*-*	*-*	*-*	no diff	Increased peak blirubin	*-*	Control group matched (GA, weight and diagnoses)
Schmidt^56^^b^	40 (fentanyl: 20)	control: 20	5–12	0.5–2	-	no (Hartwig scale)	-	-	-	*-*	*-*	*-*	*-*	*-*	*-*	-	-	*-*	*-*	*-*	*-*	-	*-*	*-*	*-*	*-*	-	No diff in gallbladder related AE	*-*	*-*
**Observational case-control study**																														
***Fentanyl high vs low dose***																														
Lammers^57^ ^X^	147 (high dose: 21)	low/no dose: 126	-	Median (IQR) cumulative dose: High 359.6 (142–1985) µg/kg Low/No dose: 0 (0–131) µg/kg	*-*	-	-	*-*	-	*-*	*-*	*-*	*-*	*-*	*-*	-	-	*-*	*-*	*-*	No association fentanyl cumulative dose and any Bayley 3 composite score at 24 months									Adjusted on multiple baseline and severity markers confounders including CRIB
***Fentanyl vs other drugs***																														
O’Mara^58^	48 (fentanyl: 24)	dexmedetomidine : 24		-	-	-	-	-	↑ adjunctive sedation compared to dex‡		-	-	increased duration compared to dex ‡	-	-	no diff (no specified timeframe)	no diff during and after drug infusion (no specified timeframe)	-	-	no diff grade III-IV IVH or PVL	-	increased time to fulle enteral compared to dex ‡	↑(9% vs 0%)	-	increased compared to dex (22% vs 11%)	-	increased compared to dex (50% vs 0%, no stats)	-	-	CRIB score balanced at baseline
Abushanab^72^^c^	126 (fentanyl: 63)	morphine: 63	0.5–3	1.0–5.0	-	PIPP	-	reduced probability of pain relief (PIPP ≤ 7) during invasive ventilation‡	-	No difference in desaturations during invasive ventilation	-	-	-	-	-	no difference (no data or timeframe)	-	-	-	-	-	-	-	-	-	-	reduced probability of analgesia failure due to withdrawal		reduced probability of analgesia failure due to death	-
**Case reports**																														
Huet^19^	1	n/a	-	1	-	-	-	-	-	_	-	↑FiO2 and PIP within 30 min of administration	-	-	-	-	-	-	-	-	-	-	-	-	-	-	-	suspected thoracic rigidity	-	NA
Lajarrige^18^	1	n/a	3	-	-	-	-	-	-	-	-	↑FiO2 and PIP within 15 min of administration	-	-	-	-	-	-	-	IVH (grade not reported)	-	-	-	-	-	-	-	suspected thoracic rigidity	Death due to IVH	NA
Pezzati^59^	1	n/a	-	1	-	-	-	-	-	-	-	-	-	-	-	-	-	-	-	-	-	paralytic ileus	-	-	-	-	-	-	-	

^X^indicates - outcomes assessed beyond neonatal period.

*Secondary studies marked in italics.*

*NA* not applicable.

†Indicates *p* < 0.05.

^a^Refers to related studies.

^b^Also in sedatives table.

^c^Also in morphine table.

**Table 4:** Studies of synthetic opioids.

Author (year)	Sample size	Comparator	Synthetic opioid dose			Analgesia			Sedation							Cardiovascular effects				Neurological effects		Gastrointestinal effect		Stress response						
	total (synthetic opioid)		Loading (µg/kg)	Continuous (µg/kg/h)	open label yes/no	Validated pain score	Reliability assessment	Analgesic efficacy		pO2/SpO2	Respiratory rate	Ventilation parameters	Duration mechanical ventilation	Bronchopulmonary dysplasia	Pneumothorax	Heat Rate	Blood pressure	Patent ductus arteriosus	Vasoactive treatment	Intraventricular Hemorrhage/Periventricular leukomalacia	Other neurological	Time to feed	Necrotizing enterocolitis		Sepsis	Renal effect urinary retention	Withdrawal	Adverse events	Mortality	Illness severity considered in analysis
***RCT studies***																														
*Remifentanil vs morphine*																														
*Pereira e Silva*^43^^a^	20 (remifentanil: 10)	Morphine: 10	1	0.5	-	NIPS and COMFORT score, before and after intubation	No	No diff between groups during infusion or in 6 h post-extubation, greater NIPS within 1hr of end infusion†	No diff in COMFORT scores	no diff	**-**	-	↓ time to extubation†	**-**	**-**	**-**	hypotension 2 vs 3 in 10 min after intubation and 4 vs 5 volume expansion (no stat)	**-**	3 vs 6 (no stat)	-	no diff neurological evaluations	no diff	-	-	-	-	-	-	-	severity of RDS balanced at baseline
*High vs low dose diamorphine*																														
*Barker*^71^	27 (high dose diamorphine: 14)	low dose diamorphine: 13	200 vs 50	15	-	-	-	-	-	↓pO2 (high vs low), ↑ PCO2 (high vs low)†				no diff	high: 1; low: 4 (no stat)		↓BP with high‡ and low dose†. No diff in infants needing dopamine (high: 4/14; low 4/13)			high: 4 vs low: 2 (no stat)	-	-	-	no diff adrenaline, noradrenaline, cortisol	-	-	-	high dose: 2/14 required resuscitation after loading	no diff (within 28 days)	-
*Diamorphine vs morphine*																														
*Wood*^44^^a^	66 (diamorphine: 44)	Morphine: 22	120 over 2 h	15	-	-	-	-	No sig diff in sedation score diamorphine vs morphine over 24 h. Shorter time to sedation - diamorphine 2 h; morphine: 6 h. Sedation adequate in 52% on diamorphine.	**-**	**-**	**-**	no diff	**-**	**-**	**-**	no sig ↓mean ABP with loading diamorphine. 32% need dopamine (vs 45% on morphine). BP variability similar (first 24 h after start).	no diff	**-**	diamorphine 52%; morphine 34% (no stat). No diff parenchymal lesions	-	-	-	Both drugs ↓adrenaline over 24 h†. No ↓noradrenaline with diamorphine.	-	-	-	-	No sig diff (diamorphine: 14%; morphine: 16%)	some cardiorespiratory indices balanced at baseline
*Alfentanil vs placebo*																														
*Saarenmaa*^65^	10 (alfentanil: 10)	Placebo (same sample; crossover design)	10 and 20	-	-	No (unvalidated behavioral pain score based on NIPS/CHEOPS) before and after painful procedures	No	↓ unvalidated score (20 µg/kg vs placebo)	-	-	-	-	-	-	-	↓HR increase (20)	no diff	**-**	**-**	**-**	**-**	-	1 (no stat)	↓adrenaline†, no diff noradrenaline	-	-	-	20µg/kg: 5/8 severe muscle rigidity	-	-
*Meperidine vs placebo*																														
*Pokela*^64^	84 (meperidine: 42)	placebo: 42	1000	-	yes	No (unvalidated behavioral pain score)	No	meperidine score < placebo score‡	-	no diff proportion of infants hypoxia. ↓ duration hypoxemia	**-**	**-**	**-**	**-**	**-**	no diff	no diff	**-**	**-**	**-**	**-**	**-**	**-**	no diff β-endorphin, cortisol, blood glucose	-	↑ urinary retention (no stat)	-	-	-	illness-related factors balanced at baseline
**Observational cohort study**																														
*Stoppa*	18 (remifentanil)	-	-	0.25 (titration)	yes	No (unvalidated score based on PIPP/Comfort) assessed during ventilation	No	Mean time to reach comfort 20 ± 13 h	-	↑ SpO2 when optimal analgesia	-	no diff MAP over time	Time to extubation 18 (3.4) min	-	-	↓ HR when optimal analgesia	-	-	-	-	-	-	-	-	-	-	-	-	-	NA
*Giannantonio*^67^	48 (remifentanil)	-	-	0.075, max 0.94	yes	NIPS and COMFORT scale	No	↓ NIPS and COMFORT at 1 h†; low scores up to 14 days	100% deep sedation (COMFORT) at 12 h	normoxia, normocapnia	-	-	Time to extubation 36 (12) min Duration MV 5.9 (5.7) days, no need for reintubation	-	-	no bradycardia	normal BP	-	-	35% IVH (<3)/0 PVL	-	No paralytic ileus, no gastric residuals	-	-	-	-	None (Finnegan)	No chest wall rigidity	3/48 (6.3%)	NA
*Elias-Jones*	34 (diamorphine)	-	50	15	-	-	-	-	-	no effect	↓ RR at 30 min, 1 h‡	-	-	-	-	↓ HR 30 min, 6 h, 12 h† (small change)	↓ BP at 30 min†	-	-	-	-	-	-	-	-	-	-	-	-	NA
*Marlow*^68^	22 (alfentanil)	-	20 or 15	3 or 5	-	-	-	-	-	transient ↓ oxygen	-	-	-	-	-	transient ↓ HR	transient ↓ BP	-	-	-	-	-	-	-	-	-	-	-	-	NA
*Pokela*^69^	20 (alfentanil)	-	9 to 15	-	-	-	-	-	-	hypoxemia 4/20 measured until 1 h after procedure	-	-	-	-	-	↓ HR 60 min	no significant change	-	-	-	No seizure, periodic activity EEG	-	-	-	-	-	-	4/20 severe muscle rigidity	-	NA
*Seguin*^70^	8 (sufentanil)	-	0.2	0.05	-	No (facial expression, cry pattern, and body movements) first 24 h	No	no signs of discomfort	-	-	-	Improvement in mechanical ventilation (increase in VEI and decrease in VI)	-	-	-	-	no hypotension	-	-	-	-	-	-	↓ β-endorphin	-	-	-	None	-	NA
**Observational case-control study**																														
Sufentanil vs phenobarbital																														
*Avenarius*^22^	38 (sufentanil: 19)	Phenobarbital: 19	0.5–2	0.5–1	yes	No (unvalidated behavioral score)	No	-	-		depression of breathing *n* = 4	-	-	-	-	-	no diff	-	↓ dopamine and dobutamine	-	-	no diff	-	-	-	-	-	2/19 thoracic rigidity	-	various diagnoses balanced at baseline
**Case reports**																														
*Pereira e Silva*^66^	1 (remifentanil)	n/a	1	0.75 for 3 h, then 0.5 for 3 h, then 0.2	-	NIPS assessed to guide dose adjustments	No	NIPS < 2, very sedated	-	-	-	-	Extubation 30 min after cessation	-	-	No bradycardia	No hypotension	-	-	-	-	-	-	-	-	No urinary retention	-	No chest wall rigidity, no laryngospasm	-	NA

†Indicates *p* < 0.05 CHEOPS—Children’s Hospital of East Ontario Pain Scale.

‡Indicates *p* < 0.01 NIPS—Neonatal Infant Pain Scale.

^a^Also in morphine table.

**Table 5:** Studies of sedatives

Author (year)	Sample size	Comparator	Sedative dose			Analgesia			Sedation	Respiratory effects						Cardiovascular effects				Neurological effects		Gastrointestinal effect		Stress response						
	total (sedative)		Loading (µg/kg)	Continuous (µg/kg/h)	open label yes/no	Validated pain score	Reliability assessment	Analgesic efficacy		pO2/SpO2	Respiratory rate	Ventilation parameters	Duration mechanical ventilation	Bronchopulmonary dysplasia	Pneumothorax	Heart Rate	Blood pressure	PDA	Vasoactive treatment	Intraventricular hemorrhage/periventricular leukomalacia	Other neurological	Time to feed	Necrotizing enterocolitis		Sepsis	Renal effect urinary retention	Withdrawal	Adverse events	Mortality	Illness severity considered in analysis
***RCT studies***																														
***Midazolam vs placebo***																														
Jacqz-Aigrain^74^	46 (midazolam: 24)	placebo: 22	-	30–60	no	-	-	-	lower scores days 1–5†	-	-	|	no difference	no difference	no difference	lower at day 1 and 2 (not day 3–5)	lower on day 1, 2, and 4‡; not on day 3 and 5; no difference hypotension (33 vs 27%)	-	midazolam 8, placebo 6	no difference	-	-	no difference	-	-	-	-	-	no difference	several illness-related factors balanced at baseline
***Midazolam vs placebo/morphine***																														
Anand^34^^a^	67 (midazolam: 22)	morphine: 24; placebo: 21	200	20–60	no	PIPP and Comfort	-	lower scores to ET suction during infusion vs placebo‡ (no specified timepoints)	no difference COMFORT during infusion (no timepoints)	-	-	-	no difference	-	no difference	-	-	-	-	no difference	higher combined IVH/PVL/death <28d† (midazolam 32 vs morphine 4 vs placebo 32%). No diff NAPI scores at 36 weeks	no difference	-	-	-	-	(mild opioid withdrawal in 2 neonates of morphine group)	-	no difference	CRIB score balanced between groups
Arya^75^	33 (midazolam + morphine: 17)	placebo + morphine: 16	200	60	no	-	-	-	better sedation scores from 18 to 48 h vs placebo/morphine† (assessed every 6 h); higher number of adequate sedation at 24, 30 and 36 h (no diff at 6, 12, 18, 42, and 48 h)	-	-	no difference during the 48 h of observation after starting infusion	no difference	-	no difference	no difference during the 48 h of observation after starting infusion	no difference during the 48 h of observation after starting infusion	-	None developed hypotension	no difference	seizures were noted in 2 neonates in placebo group 24 h after enrollment (NS)	-	-	-	-	-	-	-	-	Indication for ventilation and ventilation characteristics balanced between groups
van Alfen-van der Velden^49^^a^	21 (midazolam: 11)	morphine: 10	200	200	no	-	-	-	-	lower SaO2 between before and 15 min post-loading in midazolam group‡	no difference	-	-	-	-	no difference	lower BP between before and 15 min after loading midazolam‡; more hypotension (7/11) within 15 min after loading	-	midazolam 1, morphine 0	-	lower cerebral blood oxygenation index and cerebral blood flow velocity 15 min after loading midazolam†	-	-	-	-	-	-	increased myoclonus 5/11	-	-
***Observational cohort studies***																														
***Midazolam***																														
Jorch^79^	diazepam: 11	-	0.5	-	-	-	-	-	-	no change tcPCO2 at 5–10 min	*-*	-	-	-	-	no difference at 5–10 min	no difference MABP at 5–10 min	-	*-*	-	-	-	-	-	*-*	-	-	-	-	NA
Jacqz-Aigrain^77^	midazolam: 15	-	200 over 2–5 min	60	no	-	-	-	-	-	*-*	-	-	-	-	lower HR (4/15)	hypotension in 4/15 after the loading dose for 3/4, during infusion for 1/4	-	4 received IV albumin	-	-	-	-	-	-	-	-	Hypotension	-	NA
Harte^76^	midazolam: 10	-	100 over 2 min	-	no	-	-	-	-	no change tcPCO2 at 5,20 and 60 min	*-*	-	-	-	-	no difference at 5, 20, and 60 min	lower MBP at 5 min†, no diff from baseline at 20 and 60 min	-	None	None	lower cerebral blood flow at 5 min†, no diff from baseline at 20 and 60 min 3/10 myoclonus	-	-	-	-	-	-	3/10 Myoclonus	-	NA
Treluyer^78^	midazolam: 23	-	150–200	37.5–100	no	-	-	-	Unvalidated sedation scale: 69.4% successful sedation during suction at 1hr	no change SpO2 and oxygenation index at 1, 4, 12, 18, 24, and 48 h	*-*	No effect on triggering of ventilator breathing at 1, 4, 12, 18, 24, and 48 h	-	-	2/23 pneumothorax	Change HR: −4% (−22; 16%) in first hour	Decrease >30% mean BP in 1/23 in 48 h	-	None	-	-	-	-	-	-	-	-	none	-	NA
***Dexmedetomidine***																														
Chrysostomou^73^	dexmedetomidine: 42 (3 doses; 14/group)	-	0.05–0.2	0.05–0.2	no	NPASS	-	score > 3 during 5% of 24h infusion 17/42 patients needed more analgesia	4/42 patients needed more sedation	-	-	-	-	-	-	lower (of 12% ± 9% at 7.7 ± 7.3 h of infusion)	lower systolic BP (of 14% ± 12% at 6.5 ± 7 h)	*-*	*-*	*-*	*-*	*-*	*-*	*-*	*-*	*-*	-	3 AEs (5%) related to dexmedetomidine (no serious AEs)	-	NA
**Observational case-control studies**																														
***Midazolam***																														
Abushanab^72^	104 (midazolam + morphine: 52)	morphine: 52	100–200	10–60	yes	PIPP	-	lower successful analgesia with PIPP < 7 in 65% in morphine vs 35% in morphine + midazolam (no specified timeframe)	-	less desaturations (11 vs 26) (no specified timeframe)	-	-	increased duration (296 h vs 168 h)	no difference	-	-	-	no difference	*-*	no difference IVH	*-*	no difference	no difference	*-*	no difference	*-*	no difference	less AEs (18 vs 34)	10 vs 15	baseline characteristics included in multivariate analysis
***Dexmedetomidine***																														
O’Mara^58^^b^	48 (dexmedetomidine: 24)	fentanyl: 24	0.5 (nearly half patients)	0.3–1.2	yes	-	-	-	less adjunctive sedation during treatment period‡	-	-	-	shorter duration‡	-	-	No significant change of HR (assessed hourly during infusion)	No significant change of SBP, DBP, MBP (assessed hourly during infusion)	*-*	no difference (0 vs 0)	no difference	*-*	shorter time to full feeding†, sooner meconium†	NEC 0 vs 9% (no statistics)	*-*	less culture positive sepsis†. No difference catheter associated bloodstream infection	*-*	0 vs 50%	*-*	*-*	CRIB score balanced at baseline
***Phenobarbitone***																														
Bell^45^^a^	77 (phenobarbitone: 37)	morphine: 18; no sedation: 22	15 mg/kg	4 mg/kg/h	no	-	-	-	-	-	-	-	-	-	-	-	-	-	-	III-IV IVH: no sedation 0 vs phenobarbitone 5 vs morphine 33%	increased max burst interval on aEEG in 24 h recording after phenobarbitone or morphine administration (phenobarbitone and morphine vs no sedation). No diff phenobarbitone and morphine.	*-*	*-*	*-*	*-*	*-*	*-*	*-*	16% vs 33% (morphine) vs 0% (placebo)	-
**Case reports**																														
Reiter	Lorazepam: 1	-	5 doses of 0.3 mg/kg over 27 h, total dosage 1.5 mg/kg	-	-	-	-	-	-	lower SpO2 within 3 min after administration	-	-	-	-	-	-	-	-	-	-	Seizure at 3 min after administration EEG: suppression burst at day 0, normal at day 5	*-*	*-*	*-*	*-*	*-*	*-*	Seizures - toxic levels	*-*	NA
O’Mara^58^	Dexmedetomidine: 1	-	0.5	0.25–0.7	-	PIPP	-	elevated scores first days, then better scores and less additional medication	-	less desats during examination	-	Weaning of ventilation settings and extubation after 13 days of treatment	-	-	-	no change	MAP < 25 mmHg (hypotension prior to treatment)	-	Dopamine 10–15 mcg/kg/h	IVH grade 3	*-*	*-*	*-*	*-*	*-*	*-*	*-*	*-*	*-*	NA

†Indicates *p* < 0.05.

‡Indicates *p* < 0.01.

^a^Study also in morphine table.

^b^Study also in fentanyl table.

**Table 6:** Studies of narcotics and sedatives.

Author	Sample size	Comparator	Respiratory effects							
(year)	total (drug)				Respiratory	Ventilation	Duration mechanical		Dexamethasone	Bronchpulmonary
			pH	pO2/SpO2	rate	parameters	ventilation	Apnea	for extubation	dysplasia
*Observational - case-control studies*										
Kahn^81^	1018 (narcotics: 196)	no narcotics: 822	-	-	-	-	no diff duration MV, PPV, and O2	-	-	
Avila-alvarez	202 (analgesics or sedatives: 158)	no analgesics or sedatives: 44	-	-	-	-	-	-	-	
Toye^26^	2672 (only narcotics: 467)	no narcotics/sedatives: 1805, ony sedatives: 101, narcotics+sedatives 299	-	-	-	-	↑ duration MV (sedatives, narcotics or both)	-	-	↑ BPD (sedatives and both sedatives and narcotics)
De Tristan^80,X^	922 (narcotics and/or midazolam: 450)	no narcotics or midazolam: 472	-	-	-	-	no diff duration of MV	-	-	
Szatkowski^82^	24815 (narcotics: 20561)	no narcotics: 4254	-	-	-	-	↑ duration MV‡ (12 vs 6)	-	-	↑ BPD: 72.5% vs 60.6%

^X^indicates - outcomes assessed beyond neonatal period.

†Indicates *p* < 0.05.

‡Indicates *p* < 0.01.

### Characteristics of studies of morphine

Morphine was studied in premature infants receiving mechanical ventilation in 39 studies: 12 primary RCTs, 13 secondary reports of RCTs, 7 cohort studies, 5 case-control studies, and 2 case reports (Table 2). All studies were of intravenous administration except one^25^ in which oral morphine was included. A loading dose was administered in 19 of the 23 primary studies, ranging widely between 25 and 200 µg/kg. The most common loading dose was 100 µg/kg (12 studies). Continuous morphine was also administered in 16 primary studies at a rate ranging 5–100 µg/kg/h. Only two primary studies used infusion rates greater than 30 µg/kg/h,^28,29^ all of which were conducted in the 1990s. Six primary studies administered a maximum infusion rate of 10 µg/kg/h,^16,30,31^ and 10–30 µg/kg/h was given in a further eight studies.^25,32,33^ Five primary studies were open label, of which 4 were RCTs, and all but one study^34^ provided specific doses of rescue medication. Most studies compared morphine solely to a placebo (18/30). Other comparisons included a control group (*n* = 2); fentanyl (*n* = 2); pancuronium (*n* = 1); pancuronium or placebo (*n* = 1); diamorphine (*n* = 1); midazolam (*n* = 1); midazolam or placebo (*n* = 1); remifentanil (*n* = 1); non-standard pre-emptive morphine (*n* = 1); phenobarbitone (*n* = 1). All studies assessed outcomes within the neonatal period, except for five follow-up studies which examined neurological/neurodevelopmental outcomes^35,36^ or stress hormones in childhood.^37^ Nine of the RCTs (2 primary^38,39^ and 7 secondary) accounted for illness severity in their analyses, mostly using the Clinical Risk Index for Babies (CRIB) score.

### Morphine: analgosedation

Eleven primary studies assessed the analgesic efficacy of morphine and used a validated pain score. The most frequently used score was the Premature Infant Pain Profile (PIPP) (5/11 studies^34,38–41^); three studies used multiple different pain scores.^16,38,40^ Only one primary study assessed the reliability of this scoring.^38^ Three primary placebo RCTs reported a reduction in pain scores in response to endotracheal suction (at 2 and 12 h^16^, and 24 h^39^). Another RCT reported a significant but clinically irrelevant effect.^41^ Three trials reported no difference.^32,38,40^ Others reported no difference in analgesia compared to fentanyl^42^ or to remifentanil.^43^ Only four studies reported sedation as an outcome, three of which used COMFORT, a validated sedation score.^15,30,32,43^ One RCT compared sedation to placebo and reported a significant reduction in score at 2 and 12 h.^16^ Another RCT comparing morphine with midazolam and placebo found increased scores after stopping morphine.^34^ Two others found no difference when comparing morphine with remifentanil^43^ (during infusion or 6 h post-extubation) or diamorphine,^44^ although diamorphine induced quicker sedation.

### Morphine: risks

Higher mortality was described in three (case control studies)^33,45,46^ of 14 studies reporting mortality. One observational cohort study reported greater mortality in premature infants treated with standard morphine rather than pre-emptive morphine, but palliative patients receiving morphine were included.^31^

There was minimal evidence of adverse respiratory effects. Minor changes in ventilatory parameters were reported at various timepoints in several studies (*n* = 4; negative changes in FiO_2_; triggered breaths; functional residual capacity). Most studies reported no increase in duration of ventilation, and none reported an increase in pneumothoraces (5 placebo RCT; 2 other RCT) or bronchopulmonary dysplasia (4 placebo RCTs). There was conflicting evidence of cardiovascular effects: three placebo RCTs reported no significant difference in blood pressure,^16,30,32^ but two reported an increase in hypotension during loading and within 24 or 48 h^47,48^ and one reported lower blood pressure after the loading dose.^39^ In addition, there was no reported difference in blood pressure compared to fentanyl^42^, pancuronium^29^ or remifentanil^43^. Compared to diamorphine, lower blood pressure was reported after a loading dose^44^. Three placebo RCTs reported a small but statistically significant decrease in heart rate at time points ranging between 24 and 72 h after the start of infusion^16,30,39^. Studies reported no difference in patent ductus arteriosus.

Minimal evidence of adverse neurological effects of morphine was observed. Of the 13 RCTs that reported the incidence of intraventricular hemorrhage (IVH), only one placebo RCT reported an increase in IVH and this was specifically in infants born at 27–29 weeks of gestation.^39^ In this trial an increase in combined outcome of IVH/PVL (Periventricular leukomalacia)/death associated with use of open label morphine was identified. One study reported increased cerebral blood volume after morphine administration.^49^ Long-term neurological outcomes were assessed between 5 and 15 years in four RCT follow-up studies,^35,36^ which reported no difference in IQ, neuropsychological functioning, or thermal detection and pain thresholds. An association between opioid exposure and brain volume was reported in one study.^36^ Consistent with other studies beyond the scope of this review, suppression of brain activity, characterized by an increase in burst interval on amplitude-integrated EEG, was reported in one study compared to no sedation.^45^

There was mixed evidence of gastrointestinal effects of morphine. Of the studies that reported gastrointestinal outcomes, three reported an increased time to feed^31,39,48^ but three reported no difference.^34,42,50^ Of six studies reporting necrotizing enterocolitis (NEC) as an outcome measure, none reported an increase associated with morphine administration.^16,31,42,48,50,51^ Lastly, there was no evidence of an effect of morphine on sepsis. Urinary retention was reported in one case report,^25^ while one cohort study and two RCTs did not find an increased risk.^39,42,51^

### Morphine benefits

Apart from potential analgosedative effects, no major clinically relevant benefits were reported for morphine. One RCT observed increased mechanical ventilator synchrony in infants treated with morphine over 48 h compared to placebo.^30^ Five RCTs measured catecholamines within 24 or 96 h of starting morphine, four of which reported a significant reduction in noradrenaline^29,42,44,48^ and three of which reported a significant reduction in adrenaline.^32,42,44^

### Characteristics of studies of fentanyl

Fentanyl was the second most studied drug for analgosedation in ventilated preterm infants (Table 3). Seventeen studies were identified including nine RCTs (8 primary),^24,27,42,50,52–55^ two cohort studies,^23,56^ three case-control studies^33,57,58^ and three case reports.^18,19,59^ Fentanyl was administered intravenously in all studies. A loading dose was given in 11 primary studies, ranging from 1 to 12.5 µg/kg. A continuous infusion was administered in 13 primary studies, ranging from 0.5 to 2 µg/kg/h. The most common infusion rate was ~1 µg/kg/h. Only 1 study was open label.^27,60^ Most trials were placebo controlled RCTs (6 trials and one follow-up). Other comparators included bolus versus continuous administration,^61^ low or no dose,^57^ morphine,^33,42^ midazolam/pentobarbital,^23^ and dexmedetomidine.^58^ Only three studies assessed outcomes beyond the neonatal period.^55,57,60^ Only one RCT accounted for illness severity in their analysis. Four other RCTs confirmed no difference in CRIB score between groups at baseline.

### Fentanyl: analgosedation

Eight studies assessed the analgesic efficacy of fentanyl. Six used a validated clinical pain score. The PIPP score was most frequently used (4/6 studies^17,27,33,52^). Three studies reported multiple different pain scores.^24,27,61^ The timing of analgesic assessment ranged between 30 min after the start of infusion and 7 days. Three placebo RCTs reported significantly lower PIPP scores with fentanyl^27,52,55^; one reported no difference using the Neonatal Facial Coding System.^24^ One cohort study reported higher PIPP scores with fentanyl compared to morphine.^33^ Four studies reported sedation or adjunctive sedative use as an outcome. One placebo-controlled trial reported lower non-validated sedation scores with fentanyl,^53^ another reported low NPASS and NIPS scores with both continuous and bolus fentanyl administration.^61^ One study reported decreased adjunctive sedation compared to morphine,^23^ and another reported increased adjunctive sedation compared to dexmedetomidine.^58^

### Fentanyl: additional benefits and risks

There was no increase in mortality with fentanyl administration in the three placebo RCTs that reported this outcome. There was no clear evidence of respiratory adverse effects. Two placebo RCTs reported increased ventilatory parameters with fentanyl^27,54^ after several days of administration, whereas two reported no increase.^24,53^ Three placebo RCTs reported no difference in the duration of mechanical ventilation^27,42,53^; one trial reported slower weaning.^54^ A cohort study reported increased duration of ventilation compared to dexmedetomidine.^58^ Three placebo RCTs reported no difference in oxygenation^24,53,55^ within hours of starting infusion; three found no difference in the development of bronchopulmonary dysplasia.^27,53,54^ There was no evidence of decreased blood pressure in five placebo RCTs^24,27,42,54,55,61^ or two observational studies,^23,58^ and no difference in vasoactive treatment use in two placebo RCTs.^27,54^ Additionally, three placebo RCTs reported no difference in patent ductus arteriosus.^27,53,54^ Three placebo RCTs reported a decrease in heart rate at various time intervals that nevertheless remained within the normal range.^24,54,55^

Fentanyl was not associated with increased time to feeding (2 placebo RCTs^27,53^), sepsis (2 placebo RCTs^42,54^), urinary retention (2 placebo RCTs^27,42^) or risk of withdrawal (2 placebo RCTs,^51,53^ 1 cohort study^23^). Withdrawal was less frequent with fentanyl than with morphine in one observational study,^33^ but more frequent than with dexmedetomidine in another.^58^ Two placebo RCTs reported differences in stress-related hormones.^24,54^

There was no evidence of neurological adverse effects of fentanyl. All three placebo RCTs reporting IVH found no significant difference.^27,51,54^ In terms of neurodevelopmental outcomes, one RCT follow-up study reported a significant reduction in hand and eye coordination scores but not in developmental quotient after adjusting for confounders at 24 months.^60^ Another RCT found no difference between fentanyl and placebo for mental developmental index (MDI) and psychomotor developmental index (PDI) at 3, 6, 9, and 12 months of age.^55^ A case-control study reported no significant impact of cumulative fentanyl dose on Bayley III composite scores at 24 months after adjusting for confounders.^57^

### Studies of other synthetic opioids

A variety of synthetic opioids were studied in premature infants receiving mechanical ventilation, including remifentanil (4 studies); diamorphine (3 studies); alfentanil (3 studies); sufentanil (2 studies); and meperidine (1 study). This included five RCTs (all of which were primary), six cohort studies, one case-control study and one case report (Table 4). There were only two placebo-controlled trials, one of meperidine^62^ and one of alfentanil.^63^ All synthetic opioids were administered intravenously with infusion rates ranging as follows for different drugs: remifentanil 0.075–0.94 µg/kg/h; diamorphine 15 µg/kg/h; alfentanil 10–20 µg/kg loading dose; sufentanil 0.05–1 µg/kg/h. None of the studies investigated outcomes beyond the neonatal period. Three RCTs demonstrated a balance in various illness-related indices at baseline between groups. Studies did not account for illness severity in analyses.

### Synthetic opioids: analgosedation

There was no clear evidence of analgesic efficacy among synthetic opioids. A pain score was reported in eight studies but only three studies of remifentanil used validated pain scores (NIPS and COMFORT^43,64,65^). This included a RCT comparing remifentanil to morphine,^43^ which found no significant difference during infusion, a cohort study^65^ that reported a reduction in pain score 1-h post-administration, and a case report.^64^ An RCT of meperidine reported a significant difference in an unvalidated pain score compared to placebo.^62^ None of the studies of alfentanil^63,66,67^ or sufentanil^22,68^ assessed analgesia or sedation with a validated score. No studies of diamorphine assessed analgesia. One RCT assessed sedation with an unvalidated score and did not find a difference in sedation compared to morphine over 24 h,^44^ but reported reduced time to sedation with diamorphine. Limited evidence was available on the sedative effect of synthetic opioids. Only three studies assessed sedation, two using COMFORT,^43,65^ a validated score, to assess the effect of remifentanil. One RCT reported no difference in sedation compared to morphine^43^ and a cohort study reported deep sedation in all patients.^65^

### Synthetic opioids: additional benefits and risks

There is very little evidence for the added benefits or risks of remifentanil. In a RCT with morphine, remifentanil administration was associated with increased mean airway pressures but reduced time to extubation.^43^ There was also no difference in blood pressure or time to feed compared to morphine. No significant harms were reported. However, they did report infants developing respiratory depression,^22^ hypoxemia,^67^ severe muscle rigidity^63,67^ and thoracic rigidity.^22^ They also reported an increased incidence of IVH in infants who received diamorphine.^44^ In a RCT of high and low dose diamorphine, 2/14 infants who received high dose required resuscitation after receiving the loading dose.^69^

There were no significant changes in arterial blood pressure, heart rate, plasma-endorphin, cortisol, or glucose concentrations between meperidine and placebo.^62^

### Characteristics of studies of sedatives

Several sedative agents have been studied in premature infants receiving mechanical ventilation (Table 5). The most frequently studied sedatives were midazolam (8 studies) and dexmedetomidine (3 studies). Other agents with single studies included phenobarbitone, lorazepam, and diazepam. None of the studies investigated outcomes beyond the neonatal period. Two studies used a score to demonstrate the balance of illness severity between groups at baseline.^34,58^ One case-control study of midazolam accounted for baseline characteristics in their multivariate analysis.^70^

Midazolam was studied in 3 placebo RCTs, 1 RCT compared to morphine, 3 cohort studies and a case report. It was administered intravenously with an infusion loading dose ranging 100–200 µg, and continuous infusion rates widely ranging 20–200 µg/kg/h, with only one open label study.^70^ Only two studies used a validated pain score; one was a placebo RCT that reported significantly lower PIPP scores in response to endotracheal suction with midazolam compared to placebo.^34^ Four studies assessed sedation with midazolam; only one used a validated score. There was no difference in COMFORT scores following drug administration.^34^

Studies of dexmedetomidine included 1 case-control comparison to fentanyl, 1 dose-escalation trial and a case report. Dexmedetomidine was administered intravenously with an infusion loading dose ranging 0.05–0.5 µg and continuous infusion rates of 0.05–1.2 µg/kg/h. Only one study was open label.^58^ The primary endpoint of both the dose escalation trial and case-control study with fentanyl was the need for rescue sedation. In the dose escalation trial, premature infants were adequately sedated at all doses (based on NPASS scores and clinical judgment) and did not require additional sedatives.^71^ However, some infants (17%) did require administration of rescue analgesia. In the study comparing dexmedetomidine to fentanyl, significantly less rescue sedation and analgesia was required in patients who received dexmedetomidine.^58^

### Sedatives: additional benefits and risks

There was little evidence of neurological effects with no difference in PVL and IVH in the three placebo RCTs^34,72,73^ and two observational studies.^70,74^ However, one RCT reported an increased risk of combined IVH, PVL, or death in the midazolam group compared to morphine, but no difference in Neurobehavioral Assessment of the Preterm (NAPI) scores at 36 weeks.^34^ One RCT^49^ and one cohort study^74^ also reported decrease in cerebral blood flow with midazolam. There was no evidence of an effect of midazolam on gastrointestinal outcomes, sepsis, withdrawal, and mortality, but very few studies reported these outcomes (see table).

There was no clear evidence of respiratory effects of midazolam. The three placebo RCTs reported no significant difference in mechanical ventilation duration,^34,72,73^ O_2_ duration^72^ or ventilation parameters.^72,73^ One case-control study reported increased duration of mechanical ventilation.^70^ One placebo RCT,^72^ one RCT comparing midazolam to morphine^49^ and three cohort studies^74,75^ reported a lower BP and hypotension in the midazolam group, assessed at various timepoints ranging between 5 min and 4 days of starting the infusion. One placebo RCT did not find a difference in BP.^73^ There was mixed evidence of an effect on heart rate, with one placebo RCT^72^ and two cohort studies^75,76^ reporting reduction, whereas two RCTs^49,73^ and two cohort studies^74,77^ did not find a difference with midazolam.

For dexmedetomidine, there was very little data for added benefits and risks. In the dose escalation study, an average decrease in heart rate and blood pressure values was described and one case of diastolic hypotension was reported, none of which required intervention.^71^ In the case control study with fentanyl as comparison, shorter duration of mechanical ventilation, shorter time to full feeds and a decrease in culture positive sepsis were reported.^58^

### Studies of mixed narcotics/sedatives

We also identified studies of mixed narcotics and/or sedatives including four case-control studies and one propensity score matched cohort study (Table 6). These large studies (two retrospective and three prospective) provide an insight into outcomes related to the use of narcotics and/or sedatives versus non-exposed patients. None of them reported on analgesic or sedative efficacy of these drugs. Four studies reported on duration of mechanical ventilation, with no differences between treated or non-treated patients in two^78,79^ and an increased duration of mechanical ventilation in treated groups in the remaining.^26,80^ Only one study reported on cardiovascular outcomes with no difference in heart rate and blood pressure.^79^ 3/4 studies reported an increased incidence of severe IVH in treated groups,^26,79,80^ and one, an increased incidence of severe ROP.^26^ One study reported no difference in survival without moderate to severe neurological disabilities at 2 years.^78^ A higher incidence of death was reported by two studies^26,80^; another study reported the opposite.^78^ These conflicting results likely reflect various designs, drugs, and adjustments in these observational studies.

## Discussion

We undertook a systematic scoping review of the analgosedative agents studied in premature infants receiving mechanical ventilation to explore the benefits and risks associated with their use. Morphine, fentanyl, a variety of other synthetic opioids, and a selection of sedatives including midazolam and dexmedetomidine have been studied in this clinical context. Here, we discuss the overall benefits and risks reported for each of these drugs, identify associated gaps in our knowledge, and recommend priorities for future research.

Morphine is the most studied drug for analgosedation in ventilated preterm infants (39 studies in three decades), but its efficacy in terms of analgesia and sedation remain unclear. Morphine is considered a standard for analgosedation in children and adults; these findings lead us to question whether morphine is not as effective in this patient population, or whether it is the way it has been tested. All nine primary placebo RCTs identified in this review were conducted prior to 2014. Despite conflicting results of efficacy, over the past decade the focus has shifted to observational drug or dosing regimen comparisons and follow-up studies of the primary RCTs. Dosage of both loading boluses and continuous infusions of morphine have ranged broadly across studies. However, high doses (>100 µg/kg loading) have been particularly used in RCTs involving drug-drug comparisons, such as morphine and diamorphine,^44^ and morphine and remifentanil.^43^ Interestingly, studies which reported positive analgesic efficacy results were not studies administering the highest doses. The variability in dosage likely reflects the lack of appropriate dose-finding studies in this patient population. Furthermore, half of the placebo RCTs of morphine included open-label administration of rescue opioids complicating the assessment of analgesic efficacy. Rescue medication is an ethical imperative as infants who appear in pain cannot be ignored by the clinician. However, this non-randomized intervention can have a significant impact on the results of a trial. The administration of rescue morphine to infants receiving placebo has created an ‘as needed’ group comparison, reducing the chance of identifying a significant difference in analgesic efficacy. Equally, the administration of rescue medication to a significant proportion of infants in the morphine treatment group in several studies suggests that the drug was not providing adequate pain relief.^38,39^

Studies of morphine which reported pain outcomes used validated scores for premature infants such as PIPP, COMFORT, and NIPS. However, only two studies reported the reliability of their assessments. Given the subjective nature of these scales, adequate training, use of multiple raters, and reporting of inter- and intra-rater reliability should be conducted as standard. All studies but one^16^ assessed acute pain in response to endotracheal suctioning. Interestingly, this placebo RCT measured continuous pain (in the absence of suction) using a validated scale for premature infants (COMFORT) and reported a significant reduction in pain at 2 and 12 h.^16^ Endotracheal suctioning is a common painful^81^ procedure in NICU but given that variability in catheter size, pressure, depth, duration, and indication could potentially impact the distress and physiological instability caused by the procedure,^82^ we should question whether this non-standardized procedure is the optimal way to test analgesia during mechanical ventilation.

There is minimal data suggesting that morphine causes significant respiratory or cardiovascular adverse effects in ventilated premature infants. Some data indicated a prolonged time to establish enteral feeding,^39,50^ which could have an impact on the postnatal functional adaptation of the gut, its microbial colonization^83^ and infectious complications due to prolongation of parenteral nutrition.^84^ There were no reports of an increased incidence of NEC or sepsis. A potential increase in mortality was only reported in case-control studies. There were also no major neurological effects, except in extremely premature infants (27–29 GA), in whom intermittent boluses may be associated with an increased risk of IVH/PVL/death.^39^ Data from follow-up studies of RCTs, do not indicate long-term effects of morphine on cognitive development. However, a growing body of literature regarding the effects of cumulative morphine exposure during neonatal hospitalization, beyond the scope of this review, notably provides concerning evidence of potential long-term neurodevelopmental effects.^85^ Overall, it is difficult to identify clear benefits or risks of routine morphine administration in ventilated premature infants.

Fentanyl, the second most studied drug in ventilated premature infants, reported positive analgesic efficacy, with three of four placebo-controlled trials using validated pain scores reporting significantly lower scores following administration. However, there is little data regarding the sedative effect of fentanyl, as no placebo RCTs assessed this outcome. One study comparing bolus and continuous administration of fentanyl reported deep sedation in their participants using NPASS.^61^ Considering fentanyl is significantly more potent than morphine (50–100×) and the impact of prolonged deep sedation on the developing brain is unknown, optimal degree of sedation should be investigated in future studies. One observational cohort study compared fentanyl to morphine, but the authors used an unconventional method of assessing analgesic efficacy, limiting its utility.^33^ There is some data to suggest that an increase in ventilatory parameters may be required following administration^27,54^ but one of these studies used a larger loading dose.^54^ Reassuringly, multiple placebo RCTs reported no associated increase in the duration of mechanical ventilation. Given current concerns over potential neurological effects of opioids, it is also reassuring to note that there was no increase in IVH in the placebo RCTs, which reported this outcome. However, the only RCT that assessed later neurodevelopmental outcomes reports a poorer performance in tests of coordination and cognition at 24 months in infants who received fentanyl.^60^ Further research is needed to address optimal dosing and long-term safety of fentanyl in premature infants, particularly in infants requiring prolonged periods of mechanical ventilation. The rapid development of tolerance is a significant issue,^86^ which has not yet been addressed in this patient population and, unfortunately, may considerably limit its prolonged use in practice.

Other highly potent synthetic opioids such as remifentanil, alfentanil, and sufentanil have also been studied in preterm ventilated infants. There is limited data to assess their efficacy in this population, and no placebo-controlled trials employing a validated score to determine analgesic or sedative efficacy. The risks associated with their administration, which included reports of severe muscle rigidity and respiratory depression, clearly outweigh any potential benefits. Notably, all studies were conducted prior to 2010, and further investigations have not been undertaken likely due to the considerable risks reported. However, remifentanil and sufentanil have been studied more recently for analgosedation in term infants and in the context of surgical anesthesia and procedural analgesia, and chest wall rigidity appears to be a common and limiting adverse effect.^87–89^

Midazolam and dexmedetomidine are sedatives which have been most studied in ventilated premature infants. Given their classification as sedative drugs, it is surprising that only one RCT has assessed the sedative efficacy of midazolam in ventilated premature infants using a validated score (COMFORT), and it did not demonstrate any sedative effect.^34^ In animal models the sedative effect of midazolam is not observed until maturation of supraspinal centers; paradoxical excitation has been reported in young rats,^90^ calling into question the potential efficacy of this drug in premature infants. Clinical data on midazolam in premature infants also raise concerns over the cardiovascular and neurological effects of the benzodiazepine including hypotension, decreased cerebral blood flow, myoclonus, and increased risk of combined death/IVH/PVL in extremely premature infants. Until recently, midazolam was the most frequently used sedative in NICUs.^10^ However, with pre-clinical studies describing neuroapoptotic effects^91,92^ and clinical studies reporting potential harmful neurodevelopmental effects,^93,94^ there has been a reduction in the use of midazolam, with some countries introducing dexmedetomidine in its place.^95,96^ Dexmedetomidine is a highly selective, centrally-acting α2 adrenergic agonist, more commonly used for sedation in older children.^97^ Although there are no randomized clinical trials of dexmedetomidine in ventilated preterm infants, a stepwise dose-escalation trial of dexmedetomidine provides promising initial results in this population.^71^ None of the premature infants in the study required rescue sedative medication at any drug dose level tested, as determined by NPASS scoring/clinical judgment. However, some infants (3/18) did require administration of fentanyl as rescue analgesia. Dexmedetomidine has potential opioid sparing properties and could be efficacious as an adjunct, maximizing the efficacy of analgosedation whilst minimizing adverse effects. Encouragingly, unlike midazolam, pre-clinical data also suggest that this sedative may have neuroprotective effects,^98^ which merit further investigation in clinical trials with long-term follow-up.

In summary, we have provided an overview of the data available from studies of analgosedatives in ventilated premature infants. Overall, fentanyl appears to have the best efficacy and safety profile for analgosedation in this patient population, with a positive balance of benefits and risks. The data for morphine is less clear. Alternative synthetic opioids and midazolam are associated with significant risks in the absence of clear benefits. Dexmedetomidine may hold early promise as an opioid-sparing adjunct sedative, meriting further investigation. These results are clearly limited by the scoping nature of the review and a subsequent full systematic review with risk of bias assessment could yield further detailed conclusions.

The provision of analgosedation varies greatly worldwide and analgosedatives are no longer routinely administered to ventilated premature infants. Only ~20% of units surveyed in a recent global, prospective, cross-sectional study administer analgosedatives in more than 80% of these patients. Although opioids remain the most frequently administered agents, fentanyl use has now overtaken morphine use overall,^99^ which is encouraging given the data reviewed here. However, in England and Wales, although the use of fentanyl has increased, it remains significantly less frequently administered than morphine (fentanyl 18% vs morphine 60% of premature infants born <32 weeks).^80^ Despite NICE guidance more than half of UK units continue to routinely give morphine.^100^ Further research is required to fully establish the optimal use of fentanyl and the longer-term effects of repeated administration during extended periods of mechanical ventilation. Future studies must also take account of the impact of illness severity on clinical outcomes. To date, few studies have adjusted for illness severity in their analyses (morphine: 2 primary RCTs, 7 secondary studies; fentanyl: 1 primary RCT, 1 case-control). Given that illness severity is a key potential confounding factor, this is a significant limitation of current evidence and an important consideration for future studies.

All studies identified in this review investigated the use of pre-emptive analgosedation. Guidelines are increasingly recommending the administration of analgosedatives only ‘as required’ based on cot-side assessment of pain and sedation.^101^ This is complicated by challenges posed by inconsistent and subjective assessment of pain and distress using behaviorally focused scores. Encouragingly, most studies that used non-validated pain scores were conducted prior to 2000. Novel studies of responsive administration of analgosedatives are now needed in premature infants to justify this emerging approach to analgosedation. The rigorous use of validated objective developmentally appropriate assessments of pain will be essential.

In conclusion, based on the current data, fentanyl appears to have the most favorable efficacy and safety profile compared to morphine for use in ventilated preterm infants. Further comparative trials of responsive administration using optimal drug doses, adjunctive sedatives and long-term neurodevelopmental follow-up are needed to determine the best approach to analgosedation in this patient population.

## Supplementary information


Supplementary information


## Data Availability

The datasets generated and analyzed during the current review are available from the corresponding author on reasonable request.
