# Supplementary material for: Analgesia and sedation in premature infants receiving invasive ventilation: a systematic scoping review
Source: Pediatr Res. Author manuscript; Available in PMC 2026 Jun 11. (PMC7619142; doi:10.1038/s41390-025-04441-y)
Supplement: Supplementary [file EMS213916-supplement-Supplementary.pdf]

# Supplementary Information

## Analgesic and sedation practices in premature infants receiving invasive ventilation: a systematic scoping review

**Moultrie et al.**

## S.1. Eligibility criteria

**Table S1: Inclusion criteria**

| | |
|---|---|
| **Population** | Premature neonates undergoing invasive ventilation |
| **Intervention** | Pharmacological analgesia or sedation |
| **Comparator** | n/a |
| **Outcome** | Pain control/comfort (scores), organ systems outcomes, AEs classifications, ventilation features, parental experience |
| **Study design** | Any study design of primary empirical study (controlled, uncontrolled, interventional, observational). |
| **Report characteristics** | Primary empirical research (e.g. randomized and non-randomized controlled trials, cohort studies, case-control studies, cross-sectional studies, case series, case reports); full peer-reviewed publications; any year of dissemination; any publication language. |

**Table S2: Exclusion criteria**

| | |
|---|---|
| **Population** | Prenatal humans, neonates that are non-premature, other non-neonates. |
| **Exposure** | Non-pharmacological forms of analgesia or sedation |
| **Comparator** | |
| **Outcome** | |
| **Study design** | n/a |
| **Report characteristics** | Secondary literature (e.g. reviews, book chapters); non-empirical research (e.g. commentaries, opinions, perspectives); unpublished grey literature (e.g. conference abstracts, posters). |

## S.2. Search strategies

Embase

('prematurity'/de OR 'very low birth weight'/exp OR 'premature labor'/de OR 'neonatal intensive care unit'/de OR 'newborn intensive care'/de OR 'incubator'/de OR 'low birth weight'/exp OR (prematur* OR pre-matur* OR pre-term* OR preterm* OR ((very OR extrem*) NEAR/3 (low) NEAR/3 (birth) NEAR/3 (weight*)) OR ((low*) NEAR/3 (birthweight*)) OR VLBW OR ELBW OR LBW OR NICU OR NICUs OR ((newborn* OR neo-natal* OR neonatal*) NEAR/3 (ICU OR intensive-care*)) OR incubator* OR (born NEAR/3 (before OR 37) NEAR/3 week*) OR (weigh* NEAR/3 (1000 OR 1500 OR 2000 OR 1-000 OR 1-500 OR 2-000) NEAR/3 (gram* OR g) NEAR/3 birth) OR (very-small NEAR/3 (baby OR babies OR newborn* OR new-born*)) OR ((neurocritical) NEAR/3 (child* OR neonate*))):ab,ti,kw) **AND** ('invasive ventilation'/exp OR 'high frequency ventilation'/exp OR 'volume controlled ventilation'/de OR 'ventilated patient'/de OR 'artificial ventilation'/mj/de OR (((invasiv* OR mechanic* OR high-frequenc* OR volume-control* OR VC) NEAR/3 (ventilat*)) OR HFO):ab,ti,kw OR (((ventilat*) NEAR/3 (infant* OR preterm* OR pre-term* OR premature* OR pre-mature* OR neonate*))):ti) **AND** ('analgesic agent'/exp OR

'sedative agent'/de OR 'benzodiazepine derivative'/exp OR 'clonidine'/de OR 'propofol'/de OR 'dexmedetomidine'/de OR 'ketamine'/de OR 'pentobarbital'/de OR 'chloral hydrate'/de OR 'hydroxyzine'/de OR 'morphine derivative'/de OR 'analgesia'/de OR 'vecuronium'/de OR 'rocuronium'/de OR 'pancuronium'/de OR 'cisatracurium'/de OR (analgesic* OR analgesia OR analgetic* OR opioid* OR opiate* OR sedative* OR benzodiazepin* OR ((pain) NEAR/3 (manage* OR relief*)) OR morphine* OR fentanyl* OR fentanil* OR sufentanil* OR sufentanyl* OR alfentanil OR alfentanyl* OR remifentanil* OR remifentanyl* OR remifantan* OR midazolam* OR ketamin* OR propofol* OR dexmedetomidin* OR Cisatracurium* OR Pancuronium* OR Rocuronium* OR Succinylcholine* OR Vecuronium* OR clonidin* OR nalbuphine* OR nalbufine* OR meperidine* OR pethidine* OR codein* OR methadone* OR midazolam* OR diazepam* OR clonazepam* OR lorazepam* OR ketamine OR paracetamol* OR pentobarbital* OR chloral-hydrate* OR hydroxyzin*):ab,ti,kw) NOT ((animal/exp OR animal*:de OR nonhuman/de) NOT ('human'/exp)) NOT ([Conference Abstract]/lim OR [Conference Review]/lim) NOT 'review'/exp

Medline
(exp Infant, Premature/ OR exp Infant, Low Birth Weight/ OR exp Obstetric Labor, Premature/ OR Intensive Care Units, Neonatal/ OR exp Incubators/ OR (prematur* OR pre-matur* OR pre-term* OR preterm* OR ((very OR extrem*) ADJ3 (low) ADJ3 (birth) ADJ3 (weight*)) OR ((low*) ADJ3 (birthweight*)) OR VLBW OR ELBW OR LBW OR NICU OR NICUs OR ((newborn* OR neo-natal* OR neonatal*) ADJ3 (ICU OR intensive-care*)) OR incubator* OR (born ADJ3 (before OR 37) ADJ3 week*) OR (weigh* ADJ3 (1000 OR 1500 OR 2000 OR 1-000 OR 1-500 OR 2-000) ADJ3 (gram* OR g) ADJ3 birth) OR (very-small ADJ3 (baby OR babies OR newborn* OR new-born*)) OR ((neurocritical) ADJ3 (child* OR neonate*))).ab,ti,kf.) **AND** (exp High-Frequency Ventilation/ OR Respiration, Artificial/ OR (((invasiv* OR mechanic* OR high-frequenc* OR volume-control* OR VC) ADJ3 (ventilat*)) OR HFO).ab,ti,kf. OR (((ventilat*) ADJ3 (infant* OR preterm* OR pre-term* OR premature* OR pre-mature* OR neonate*))).ti.) **AND** (exp Analgesics/ OR exp Analgesics, Opioid/ OR Hypnotics and Sedatives/ OR exp Benzodiazepines/ OR Clonidine/ OR Propofol/ OR Dexmedetomidine/ OR Ketamine/ OR Pentobarbital/ OR Chloral Hydrate/ OR exp Hydroxyzine/ OR exp Morphine Derivatives/ OR Analgesia/ OR Vecuronium Bromide/ OR Rocuronium/ OR Pancuronium/ OR cisatracurium.nm. OR (analgesic* OR analgesia OR analgetic* OR opioid* OR opiate* OR sedative* OR benzodiazepin* OR ((pain) ADJ3 (manage* OR relief*)) OR morphine* OR fentanyl* OR fentanil* OR sufentanil* OR sufentanyl* OR alfentanil OR alfentanyl* OR remifentanil* OR remifentanyl* OR remifantan* OR midazolam* OR ketamin* OR propofol* OR dexmedetomidin* OR Cisatracurium* OR Pancuronium* OR Rocuronium* OR Succinylcholine* OR Vecuronium* OR clonidin* OR nalbuphine* OR nalbufine* OR meperidine* OR pethidine* OR codein* OR methadone* OR midazolam* OR diazepam* OR clonazepam* OR lorazepam* OR ketamine OR paracetamol* OR pentobarbital* OR chloral-hydrate* OR hydroxyzin*).ab,ti,kf.) NOT (exp Animals/ NOT Humans/) NOT (news OR congres* OR abstract* OR book* OR chapter* OR dissertation abstract*).pt. NOT (Systematic Review/ OR Review/)

Cochrane
((prematur* OR pre NEXT matur* OR pre NEXT term* OR preterm* OR ((very OR extrem*) NEAR/3 (low) NEAR/3 (birth) NEAR/3 (weight*)) OR ((low*) NEAR/3 (birthweight*)) OR VLBW OR ELBW OR LBW OR NICU OR NICUs OR ((newborn* OR neo NEXT natal* OR neonatal*) NEAR/3 (ICU OR intensive NEXT care*)) OR incubator* OR (born NEAR/3 (before

OR 37) NEAR/3 week*) OR (weigh* NEAR/3 (1000 OR 1500 OR 2000 OR 1 NEXT 000 OR 1 NEXT 500 OR 2 NEXT 000) NEAR/3 (gram* OR g) NEAR/3 birth) OR (very NEXT small NEAR/3 (baby OR babies OR newborn* OR new NEXT born*)) OR ((neurocritical) NEAR/3 (child* OR neonate*))):ab,ti,kw) **AND** ((((invasiv* OR mechanic* OR high NEXT frequenc* OR volume NEXT control* OR VC) NEAR/3 (ventilat*)) OR HFO):ab,ti,kw OR (((ventilat*) NEAR/3 (infant* OR preterm* OR pre NEXT term* OR premature* OR pre NEXT mature* OR neonate*))):ti) **AND** ((analgesic* OR analgesia OR analgetic* OR opioid* OR opiate* OR sedative* OR benzodiazepin* OR ((pain) NEAR/3 (manage* OR relief*)) OR morphine* OR fentanyl* OR fentanil* OR sufentanil* OR sufentanyl* OR alfentanil OR alfentanyl* OR remifentanil* OR remifentanyl* OR remifantan* OR midazolam* OR ketamin* OR propofol* OR dexmedetomidin* OR Cisatracurium* OR Pancuronium* OR Rocuronium* OR Succinylcholine* OR Vecuronium* OR clonidin* OR nalbuphine* OR nalbufine* OR meperidine* OR pethidine* OR codein* OR methadone* OR midazolam* OR diazepam* OR clonazepam* OR lorazepam* OR ketamine OR paracetamol* OR pentobarbital* OR chloral NEXT hydrate* OR hydroxyzin*):ab,ti,kw) NOT review:pt

<u>Web of Science</u>
TS=(((prematur* OR pre-matur* OR pre-term* OR preterm* OR ((very OR extrem*) NEAR/2 (low) NEAR/2 (birth) NEAR/2 (weight*)) OR ((low*) NEAR/2 (birthweight*)) OR VLBW OR ELBW OR LBW OR NICU OR NICUs OR ((newborn* OR neo-natal* OR neonatal*) NEAR/2 (ICU OR intensive-care*)) OR incubator* OR (born NEAR/2 (before OR 37) NEAR/2 week*) OR (weigh* NEAR/2 (1000 OR 1500 OR 2000 OR 1-000 OR 1-500 OR 2-000) NEAR/2 (gram* OR g) NEAR/2 birth) OR (very-small NEAR/2 (baby OR babies OR newborn* OR new-born*)) OR ((neurocritical) NEAR/2 (child* OR neonate*)))) **AND** ((((invasiv* OR mechanic* OR high-frequenc* OR volume-control* OR VC) NEAR/2 (ventilat*)) OR HFO) OR (((ventilat*) NEAR/2 (infant* OR preterm* OR pre-term* OR premature* OR pre-mature* OR neonate*))):ti) **AND** ((analgesic* OR analgesia OR analgetic* OR opioid* OR opiate* OR sedative* OR benzodiazepin* OR ((pain) NEAR/2 (manage* OR relief*)) OR morphine* OR fentanyl* OR fentanil* OR sufentanil* OR sufentanyl* OR alfentanil OR alfentanyl* OR remifentanil* OR remifentanyl* OR remifantan* OR midazolam* OR ketamin* OR propofol* OR dexmedetomidin* OR Cisatracurium* OR Pancuronium* OR Rocuronium* OR Succinylcholine* OR Vecuronium* OR clonidin* OR nalbuphine* OR nalbufine* OR meperidine* OR pethidine* OR codein* OR methadone* OR midazolam* OR diazepam* OR clonazepam* OR lorazepam* OR ketamine OR paracetamol* OR pentobarbital* OR chloral-hydrate* OR hydroxyzin*)) NOT ((animal* OR rat OR rats OR mouse OR mice OR murine OR dog OR dogs OR canine OR cat OR cats OR feline OR rabbit OR cow OR cows OR bovine OR rodent* OR sheep OR ovine OR pig OR swine OR porcine OR veterinar* OR chick* OR zebrafish* OR baboon* OR nonhuman* OR primate* OR cattle* OR goose OR geese OR duck OR macaque* OR avian* OR bird* OR fish*) NOT (human* OR patient* OR women OR woman OR men OR man))) AND DT=(Article OR Letter OR Early Access)

<u>Google Scholar (-review in title)</u>
premature|"pre-mature"|"pre-term"|preterm|"very|extremely low birth weight"|VLBW|ELBW|LBW
ventilation|ventilated
analgesic|analgesia|analgetic|opioid|opiate|sedative|benzodiazepine|morphine|fentanyl|midazolam|ketamin|propofol|clonidin|codein

**S.3. Data items**

**A- Study characteristics**

1. Design of the study
   Study design terminology used in this review is defined in Grimes and Schulz, 2002 (https://doi.org/10.1016/S0140-6736(02)07283-5)
2. Continent/Country where the study was conducted
3. Publication year
4. Site characteristics (single/multiple centers)
5. Primary outcomes

**B- Patients' characteristics**

(Age terminology used in this review is defined according to the American Academy of Pediatrics (https://doi.org/10.1542/peds.2004-1915))

6. Gestational age at birth
7. Birth weight
8. Postnatal age at time of study
9. Postmenstrual age at time of study
10. Weight at inclusion

**C- Interventions and outcomes**

11. Drugs (types, dose, duration, administration type, route)
12. Pain /comfort score used for intervention
13. Pain /comfort score used as outcome measure
14. Persons who assessed pain/comfort scores (nurses, doctors, parents, researchers)
15. Efficacy based on pain/comfort scores
16. Respiratory outcomes (e.g. BPD, pneumothorax, VAP, change in PCO2, duration of MV)
17. Circulatory outcomes (e.g. blood pressure, PDA, urine output)
18. Digestive outcome (e.g. spontaneous intestinal perforation, NEC, time to full-feed, duration of parenteral nutrition)
19. Neurological outcomes (e.g. IVH, PVL, CP, neurodevelopmental scores, visual outcomes, auditory outcomes)
20. Infections (e.g. secondary infections except NEC and VAP)
21. Assessment of MV parameters used (e.g. conventional ventilation or HFOV, mean airway pressure, FiO2, assessment of MV synchrony, duration of NIV, others?)
22. Adverse events
23. Mortality
24. Parental experience
